# Screening key sorghum germplasms for low-nitrogen tolerance at the seedling stage and identifying from the carbon and nitrogen metabolism

**DOI:** 10.3389/fpls.2024.1340509

**Published:** 2024-09-12

**Authors:** Chunjuan Liu, Wendong Gu, Bang Li, Yihao Feng, Chang Liu, Xiaolong Shi, Yufei Zhou

**Affiliations:** ^1^ College of Agronomy, Shenyang Agricultural University, Shenyang, Liaoning, China; ^2^ College of Agronomy and Horticulture, Liaoning Agricultural Vocational and Technical College, Yingkou, Liaoning, China

**Keywords:** *Sorghum bicolor*, low-N tolerance screening, genotypic variation, phenotype, C metabolism, N metabolism

## Abstract

**Introduction:**

Sorghum (*Sorghum bicolor* L.) can withstand drought and heat stress and efficiently utilize water and nutrients. However, the underlying mechanism of its tolerance to low-nitrogen (N) stress remains poorly understood.

**Materials and methods:**

This study assessed low-N tolerance in 100 sorghum-inbred lines and identified those with exceptional resilience. Principal component analysis, Pearson’s correlation, and Y value analysis were used to examine various seedling growth metrics, including plant and root dimensions, biomass, chlorophyll content, root N content, shoot N content, and root/shoot ratio.

**Results and discussion:**

The genotypes were categorized into four distinct groups based on their respective Y values, revealing a spectrum from highly tolerant to sensitive. Low-N-tolerant sorghum lines maintained higher photosynthetic rates and exhibited increased enzymatic activities linked to carbon and N metabolism in the leaves and roots. Furthermore, low-N-tolerant genotypes had higher levels of key amino acids, including cystine, glycine, histidine, isoleucine, leucine, phenylalanine, threonine, and tyrosine, indicating a robust internal metabolic response to N deficiency.

**Conclusion:**

This study provides a comprehensive and reliable approach for the evaluation of sorghum tolerance to low-N environments, sheds light on its morphological and physiological adaptations, and provides valuable insights for future breeding programs and agricultural practices.

## 1 Introduction

Nitrogen (N) is essential for crop growth, production, and sustainable agricultural development (Spiertz, 2010; Ren et al., 2022). However, the efficacy of nitrogen fertilizers in the field is strikingly low, with less than half of the applied N being absorbed by crops (Lassaletta et al., 2014). Consequently, the Chinese government has made concerted efforts in recent years to improve agricultural ecosystems by reducing excessive fertilizer input and implementing effective tillage practices. Despite these commendable endeavors, the issue of nitrogen underutilization persists, which underscores the urgent need for crop cultivars with optimized N-use efficiency that will be able to thrive with reduced fertilizer inputs and thus align with the principles of sustainable agriculture. Previous studies have shown that low-N-tolerant genotypes can produce favorable results (Liu et al., 2020) and that N deficiency may impede plant health, affecting various biological levels from tissues to molecules (Qin et al., 2019; Liu et al., 2022b; Wang et al., 2023). Extensive expanses of barren and currently underutilized land hold considerable potential as reserve areas for agricultural production. Prudent exploitation can alleviate the strain on existing arable land; however, the existing challenge of soil nitrogen deficiency must first be overcome. Consequently, the mitigation of the harmful effects of soil N deficiency has attracted the attention of the research community and stakeholders, highlighting the importance of this research in the broader context of agricultural and environmental health.

To overcome soil N deficiency, plants employ a suite of morphological and physiological responses, including changes in biomass, root architecture, N uptake and translocation, and N and carbon (C) metabolism (Schlüter et al., 2012; Wang et al., 2018b). Among these adaptations, root architecture modifications are particularly crucial to increase plant N uptake under low-N conditions (Gao et al., 2015; Li et al., 2016). In maize (*Zea mays* L.), the “steep, cheap, and deep” root phenotype extends root depth to optimize N capture and improve the acquisition of N resources from deep soil domains under limited N availability (Lynch and Wojciechowski, 2015; Schäfer et al., 2022). Furthermore, N deficiencies often manifest as visible changes in leaf color, with shifts from green to yellow due to reductions in chlorophyll content, which is an apparent phenomenon across various plant species under low-N stress (Luo et al., 2019; Zhong et al., 2019). Hirel et al. (2005) conducted field experiments and found that N deficiency decreased the chlorophyll, total amino acid, and protein content of maize leaves. N deficiency also affects plant leaf chlorophyll fluorescence, light energy distribution, and photosynthetic capacity—for example, N deficiency reduced the maximum efficiency of PSII photochemistry (Fv/Fm), electron transport rate (ETR), non-photochemical chlorophyll fluorescence quenching (NPQ), photochemical quenching (qP), photosynthetically active radiation (PAR), intercepted photosynthetically active radiation (IPAR), and radiation use efficiency (RUE) in plants (Hikosaka, 2014; Liu et al., 2021). This impact is associated with the fact that N assimilation requires energy in the form of ATP and NADPH as well as C-skeletons (C-skeleton is an important component of synthetic organic compounds) derived from photosynthesis in illuminated leaves—for instance, an increase in starch accumulation under low-N conditions can be partially attributed to the diminished demand of C-skeletons for amino acid assimilation (Schlüter et al., 2012). Due to the pivotal role of N in plant growth and physiology, genotype screening, confirmation of indicators of low-N tolerance, and further elucidation of the physiological mechanisms underlying low-N tolerance crops are essential. These insights will facilitate the improvement of breeding practices and inform the development of optimal management strategies for the cultivation of infertile lands.

Sorghum (*Sorghum bicolor* L.), known for its tolerance to drought, high salinity, and heat stress, is extensively cultivated in arid and marginal regions of the world (Farré and Faci, 2006; Liu et al., 2024). This robust crop surpasses its grassy counterparts in terms of its higher water and nutrient use efficiency, making it an excellent candidate for sustainable agricultural practices (Paterson et al., 2009; Ngara and Ndimba, 2014; Carcedo et al., 2022). Buchanan et al. (2005) showed that a deep root system is a crucial factor in sorghum drought tolerance and significantly enhances its water use efficiency. Addy et al. (2010) discovered that stay-green sorghum had enhanced N use and uptake, indicating the potential for improved nitrogen management within the species. Despite these advances, the specific genotypes of sorghum with low-N tolerance have not been well documented. The identification and cultivation of low-N-tolerant sorghum genotypes present an opportunity to harness this crop’s potential for low-N-affected soil, promising both economic and resource-saving benefits.

This study screened and evaluated low-N tolerance in 100 sorghum inbred lines. Their physiological changes were studied in response to low-N stress during the seedling stages to determine their morphological (plant height and root characteristics) and physiological (SPAD, root N content, and shoot N content) attributes. The genotypes were then categorized as tolerant or sensitive to low-N conditions. Seedling growth performance of inbred lines was evaluated to determine the physiological characteristics that define sorghum adaptability. This study provides a theoretical foundation for the strategic utilization and improvement of sorghum cultivation in infertile soils. In addition, the results will help to improve the environmental impact of conventional agricultural practices.

## 2 Materials and methods

### 2.1 Plant (*Sorghum*) materials

A total of 100 sorghum inbred lines were used in this study. The plant names are listed in **Supplementary Table S1**. All sorghum plants were grown under hydroponic conditions at Shenyang Agricultural University, Shenyang, Liaoning, China.

### 2.2 Hydroponic growth conditions

Hydroponic experiments were conducted in a laboratory climate chamber. The plants were grown in a hydroponic box (0.4 × 0.25 × 0.2 m). Sorghum seeds of the same size and full grain were selected for sterilization with 10% NaClO solution for 5–10 min, washed with distilled water, placed in Petri dishes covered with wet filter paper, and cultured in an incubator under the following conditions: day/night temperatures of 28°C/25°C and light/dark periods of 12 h. After 3 days, seedlings were selected and transferred to 1/2 Hoagland nutrient solution, which (as control, normal N [NN]) consisted of 3 mM KNO_3_, 0.5 mM NH_4_H_2_PO_4_, 1 mM MgSO_4_·7H_2_O, 2 mM Ca(NO_3_)_2_·4H_2_O, 0.05 M Fe-EDTA, 5 × 10^-3^ mM MnCl_2_·4H_2_O, 2.5 × 10^-4^ mM H_3_BO_3_, 0.5 × 10^-3^ mM ZnSO_4_·7H_2_O, 0.2 × 10^-3^ mM CuSO_4_·5H_2_O, and 0.1 × 10^-3^ mM (NH_4_)_6_Mo_7_O_24_·4H_2_O. After the third leaf stage, the seedlings were treated with NN or low-N (LN: 0.05 mmol·L^-1^ N). The composition of the other plant nutrients remained unchanged during low-N treatment. The low-N treatment was performed for 10 days, after which the seedlings were harvested (four biological replicates). Shoot and root tissues were separated, and samples from each group were mixed in replicates. The leaves and roots were sampled for physiological analysis, frozen in liquid N, and stored at −80°C.

### 2.3 Agronomic traits

Five sorghum seedlings were selected from each experimental treatment. The absolute distance from the base of the stem to the longest leaf in the upper part of the stem (plant height) and that from the base of the plant to the lowest part of the root (root length) were measured. For each replicate, three randomly selected seedling leaves and roots were analyzed.

The plants were separated into roots and shoots and dried in an oven at 70°C until they reached a constant weight. The dry weights of shoots and roots were determined using an electronic balance with a precision of 0.001 g.

### 2.4 Leaf and root microstructure

Leaf and root tip microstructures were prepared in accordance with the methodology described by Qi et al. (2014) to measure leaf and root cell structures. In brief, 15–20-mm leaf and 3-μm root tip transections were prepared and fixed in a fixing solution. The fixed leaves and roots were dehydrated, embedded, sliced, and stained for 2 h. Subsequently, they were decolorized with different alcohol gradients, dyed with solid green, and sealed with transparent xylene and neutral gum. The slices were scanned and processed using a scanner (Pannoramic DESK, P-MIDI, P250, Hungary) and scanning software (Pannoramic Scanner) to count the number of cortex cells in the meristematic zone and calculate the length of cells in the elongation zone of root tips.

### 2.5 Leaf area and chlorophyll content (SPAD value)

A total of 12 sorghum seedling leaves were used in each treatment to quantify the leaf area, with the latter determined using length and width measurements. For each replicate, four sorghum seedling leaves were randomly selected and analyzed three times. A total of 12 sorghum seedling leaves were randomly selected from each treatment and used to measure the leaf chlorophyll content. Sorghum seedling leaves were sampled using a hole punch (diameter, 1.2 mm). After sampling, the leaves were placed in an opaque reagent bottle containing 10 mL of 80% acetone solution and kept in the dark for 24 h to extract the total chlorophyll content. The total chlorophyll content was determined to be 645 nm as described by Liu et al. (2022a).

SPAD values were determined using a portable SPAD-502 (Konica Minolta, Inc., Tokyo, Japan). Measurements were conducted five times for each leaf, and the mean value was calculated as the SPAD value for a given leaf. At least four readings were obtained for each treatment in each plot.

### 2.6 Leaf photosynthetic parameter

The leaf net photosynthetic rate (Pn), stomatal conductivity (Gs), transpiration rate (Tr), and intercellular CO_2_ concentration (Ci) of the first fully expanded sorghum leaf were determined using Li-6400 (LI-COR, USA), and photosynthesis-related indices were determined four times for each treatment. The parameters were set as follows: light intensity, 1,200 μmol m^-2^ s^-1^; CO_2_ concentration, 500 ± 5 μmol mol^-1^.

### 2.7 Leaf amino acid seed content

Sorghum seedling leaves were digested with 1 mL of 6 M HCl at 110°C for 24 h. Next, 1 mL of the sample was centrifuged at 15,000 ×*g* for 10 min. The supernatant was neutralized using 2 M NaOH. The neutralized and diluted samples were subjected to ultra-performance liquid chromatography (UPLC) coupled to a Vanquish mass spectrometer (MS) equipped with a quadrupole electrospray ionization (QE) source. The analytical conditions were as follows: a C18 column (1.7 μm, 50 × 2.1 mm) was maintained at 55°C with a flow rate of 0.5 mL/min and injection volume of 1 μL. Gradient elution was then performed. Finally, the data were analyzed using LC–MS/MS on a Q Exactive hybrid Q–Orbitrap mass spectrometer equipped with a heated ESI source (Thermo Fisher Scientific) using the full MS acquisition method (Glauser et al., 2016).

### 2.8 Leaf N, nitrate (NO_3_^-^), and ammonium (NH_4_^+^) content

The leaves and roots were dried, pulverized, and boiled in concentrated sulfuric acid. Total N content was determined using the Kjeldahl method. The nitrate and ammonium contents in the roots were determined using colorimetric and ninhydrin colorimetric methods, respectively, with some modifications (Wang et al., 2018a). Briefly, fresh root (0.1 g) was ground into a homogenate with distilled water, transferred to a centrifuge tube, and boiled for 10 min. Subsequently, the supernatant was centrifuged at 15,000 ×*g* for 10 min, and 0.1 mL of the supernatant was added to 0.4 mL of 5% salicylic-sulfuric acid solution and reacted at 20°C under light for 20 min. Furthermore, 9.5 mL of 8% NaOH solution was added slowly. Finally, the NO_3_^-^ content was quantified at 410 nm using a spectrophotometer.

Similarly, roots (0.1 g) were placed in 10% acetate to extract NH_4_^+^, mixed with distilled water, and filtered. The supernatant was combined with a solution of ninhydrin reagent (ninhydrin with propanol, butyl alcohol, glycol, and acetate, pH 5.4) and 1% ascorbic acid and boiled for 15 min. The concentration of NH_4_^+^ was quantified at 580 nm.

### 2.9 Leaf and root N-metabolism enzyme activities

Nitrate reductase (NR) and nitrite reductase (NiR) activities were determined using nitrate as described by Qu et al. (2023). Briefly, samples (200 mg) were ground in an extraction buffer containing potassium phosphate (pH 8.8), EDTA, cysteine, and 3% (w/v) bovine serum albumin (BSA). The absorbance values of the NR and NiR supernatants were measured at 540 nm.

Glutamine synthetase (GS) and glutamate synthase (GOGAT) activity levels were analyzed using methods described by Wang et al. (2018a) and Luo et al. (2013). Briefly, leaves or roots (200 mg) were extracted in buffer (Tris-HCl, pH 8.0; MgSO_4_, DTT, and sucrose) to measure GS activity. In addition, the leaves and roots were extracted with Tris-HCl (pH 7.6), MgC1_2_, EDTA, and β-mercaptoethanol at 4°C to measure GOGAT activity. After the homogenate was centrifuged at 15,000 × *g* for 30 min at 4°C, the absorbance of the GS and GOGAT supernatants was measured at 540 and 340 nm, respectively.

### 2.10 Leaf and root relative C content

The soluble sugar, sucrose, and starch contents were determined by anthrone sulfate colorimetry (Ren et al., 2023). Fresh leaf and root samples (0.1 g) were extracted using 80% ethanol solution. The supernatant was collected, and its volume was fixed at 20 mL with 80% ethanol to determine the soluble sugar and sucrose contents. The starch content was determined by precipitation.


(1) $$\begin{array}{c} Total\;carbon\;\left(C\right)\;content\;\left(mg/g\right)\\ \;=soluble\;sugar\;content+starch\;content \end{array}$$

### 2.11 Leaf and root relative C metabolism enzyme activities

The leaves and roots were extracted in a buffer (Tris-HCl, β-mercaptoethanol, MgCl_2_, ethylene glycol-bis-β-aminoethyl ether, ethylenediaminetetraacetic acid, and 15% glycerol). The mixed solutions were centrifuged for 15 min at 17,000 ×*g* (4°C). The supernatant was used to determine enzyme activity. The activities of sucrose phosphate synthase (SPS), sucrose synthase (SuSy), and invertase (INV) were measured as described by Shahid et al. (2019).

### 2.12 Data analysis

Data from at least three replicates are presented as mean ± SE. Analysis of variance (ANOVA) was performed using the Duncan test design method with SPSS software (version 19.0, IBM, USA) at probability levels of 5%, 1%, and 0.1% for each data point with at least three duplications. Graphics were created using GraphPad Prism 8.3.0 and Adobe Photoshop 2020.

## 3 Results

### 3.1 Comprehensive evaluation of low-N tolerance in 100 sorghum inbred lines

#### 3.1.1 Analysis of low-N tolerance coefficients

**Figure 1:**
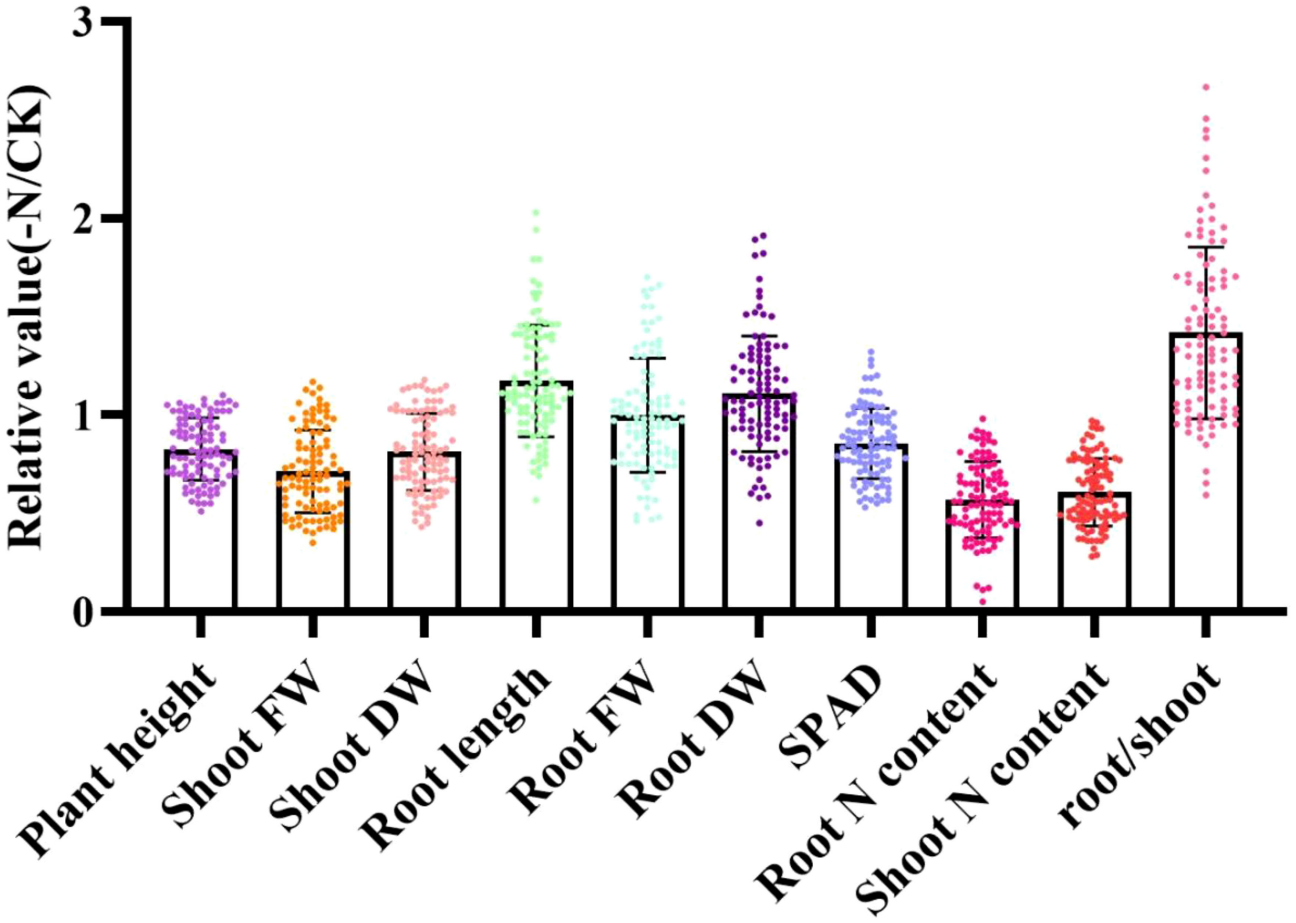
Relative morphological and physiological property values conveying tolerance to N stress in 100 sorghum genotypes determined using a hydroponic experiment. Data are expressed relative to the treatment with normal nitrogen (NN: 7.5 mmol·L^-1^ N). FW, fresh weight; DW, dry weight; plant height (cm); root length (cm); shoot FW (g); shoot DW (g); root FW (g); root DW (g); root N content (g/kg); and shoot N content (g/kg). The X axes represent the relative values, which were the values under low-N treatment (-N) compared with the values under normal N treatment (CK). The Y axes represent the morphological and physiological parameters.

To eliminate inherent biological differences between genotypes, the relative value (value of each index under low N stress/value of each index under normal N stress) was used to characterize low-N tolerance during the sorghum seedling period (**Figure 1**). Under low-N stress, the ranges of variation in relative plant height and root length were 0.51–1.10 and 0.57–2.03, respectively (**Supplementary Table S2**). Similarly, the ranges in variation in the relative shoot fresh weight, shoot dry weight, root fresh weight, root dry weight, root N content, shoot N content, and root/shoot ratio were 0.35–1.17, 0.43–1.18, 0.46–1.70, 0.45–1.91, 0.05–0.98, 0.28–0.97, and 0.59–2.67, respectively, and most genotypes had proportionate root and shoot ratios >1. These data indicate that the effects of N stress on root growth were greater than those on leaf growth.

#### 3.1.2 Pearson’s correlation analysis of low-N tolerance coefficients

**Figure 2:**
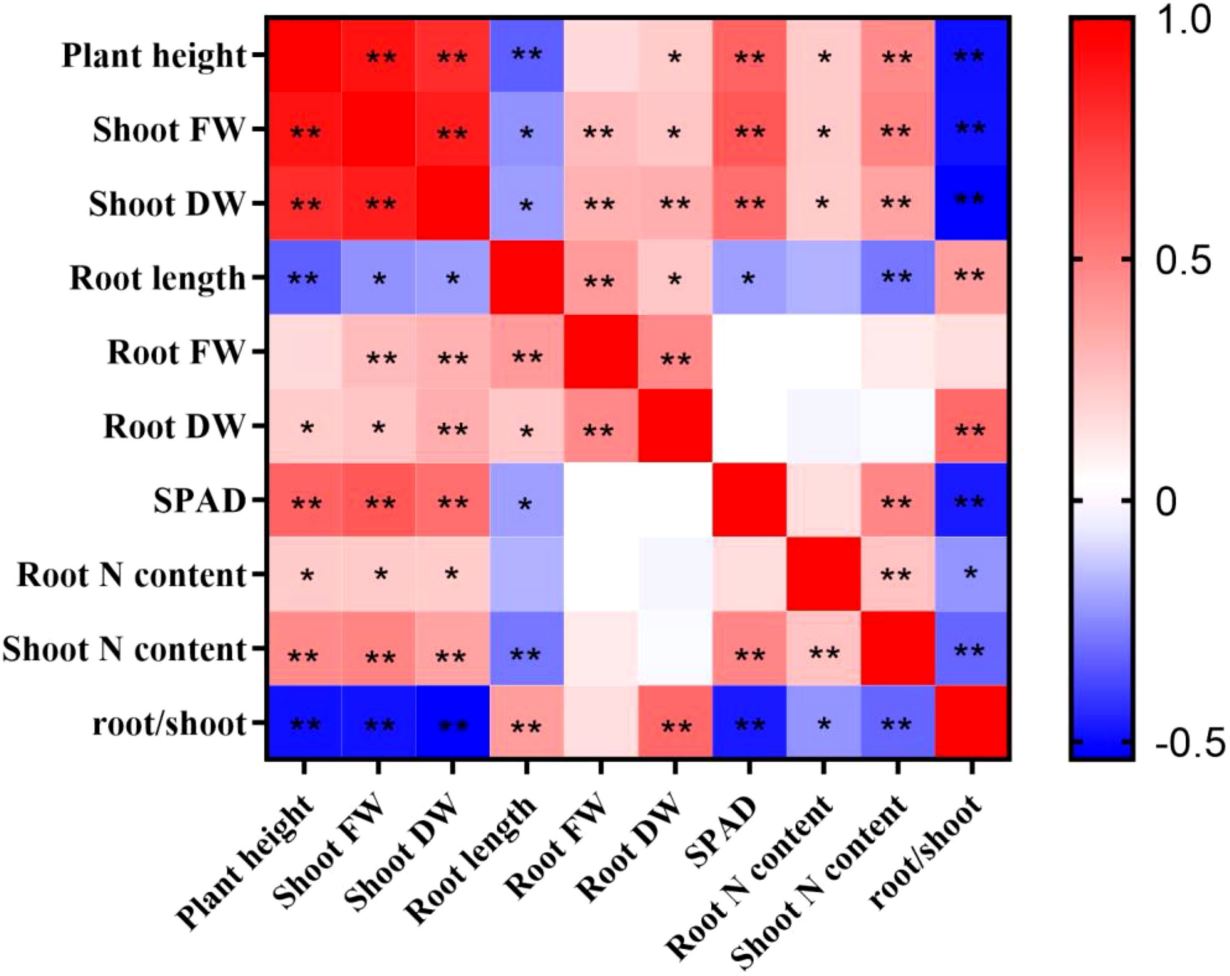
Pearson’s correlation coefficients for the sorghum traits after 10 days of normal-N (NN) and low-N (LN) treatment. FW, fresh weight; DW, dry weight. * and ** indicate significant correlations of 0.05 and 0.01, respectively.

To gain a more comprehensive understanding of the low-N tolerance coefficients, Pearson’s correlation analysis was conducted (**Figure 2**). Plant height, shoot fresh weight, shoot dry weight, root fresh weight, root dry weight, SPAD, root N content, and shoot N content exhibited a positive correlation with each other. Root length was significantly negatively correlated with plant height, shoot fresh weight, shoot dry weight, SPAD, root N content, and shoot N content. Similarly, the root/shoot ratio was also significantly negatively correlated with plant height, shoot fresh weight, shoot dry weight, SPAD, and root and shoot N content. In contrast, root length was significantly positively correlated with the root/shoot ratio, indicating that low-N stress promoted sorghum root growth. Nevertheless, there may be some overlap between agronomic and physiological indicators. This indicates that comprehensive and variable indicators can be used to screen and evaluate low-N-tolerant sorghum germplasm resources.

#### 3.1.3 Principal component analysis of low-N tolerance coefficients

Dimensionality reduction analysis can effectively eliminate complex factors with greater interference and less impact. Consequently, PCA was used to enhance the precision of the parameter measurement analysis. PCA transformed low-N tolerance coefficients (indicators) into more effective and lower index numbers in accordance with the low-N tolerance coefficients of the 100 sorghum genotypes. As illustrated in **Tables 1** and **2**, PCA was conducted using the afore-mentioned indicators, resulting in the division of data into three principal components, with a cumulative contribution of 81.03%. In the first principal component, the eigenvalue and largest contribution rate (root/shoot ratio) were 0.289 and 44.21%, respectively. Subsequently, the eigenvalue and second contribution rate (root fresh weight) were 0.159 and 24.62%, respectively. The third principal component, with an eigenvalue of 0.079 and a contribution rate of 12.12%, is the root length. The first three principal components were sufficient to explain the changes in the data, thus meeting the PCA requirements. Consequently, the low-N tolerance characteristics of sorghum can be objectively determined using three independent comprehensive indicators.

**Table 1:** Principal component eigenvalues and their contribution rates.

Principal component	Eigenvalue	Contribution (%)	Cumulative contribution (%)
1	0.286	44.214	44.214
2	0.159	24.623	68.836
3	0.079	12.193	81.030

**Table 2:** Loading matrix of each component.

Traits (%)	Principal component		
	1	2	3
Plant height	0.089	0.103	0.041
Shoot fresh weight	0.114	0.156	0.033
Shoot dry weight	0.106	0.15	0.019
Root length	-0.159	0.047	-0.199
Root fresh weight	-0.075	0.216	-0.125
Root dry weight	-0.166	0.209	0.099
SPAD	0.098	-0.073	0.022
Root N content	-0.072	-0.058	0.031
Shoot N content	0.055	-0.031	0.02
Root/shoot	-0.422	-0.006	0.094

#### 3.1.4 Comprehensive evaluation of low-N tolerance coefficients

Based on the PCA results, a comprehensive analysis of all sorghum genotypes was performed and then used in the Y value analysis. According to the score coefficient matrix for each factor (**Supplementary Table S3**), the original 10 indicators were converted into three principal components, and the obtained formula were as follows:


(2) $$\begin{array}{c} Y1=0.563X1+0.543X2+0.539X3-0.563X4-0.257X5\\ \;-0.563X6+0.544X7+0.417X8+0.282X9\\ \;-0.968X10 \end{array}$$

(3) $$\begin{array}{c} Y2=0.652X1+0.740X2+0.762X3+0.165X4+0.744X5\\ \;+0.709X6+0.408X7+0.336X8+0.160X9\\ \;+0.012X10 \end{array}$$

(4) $$\begin{array}{c} Y3=0.260X1+0.156X2+0.098X3-0.703X4-0.431X5\\ \;+0.336X6+0.121X7+0.179X8+0.105X9\\ \;+0.215X10 \end{array}$$

**Figure 3:**
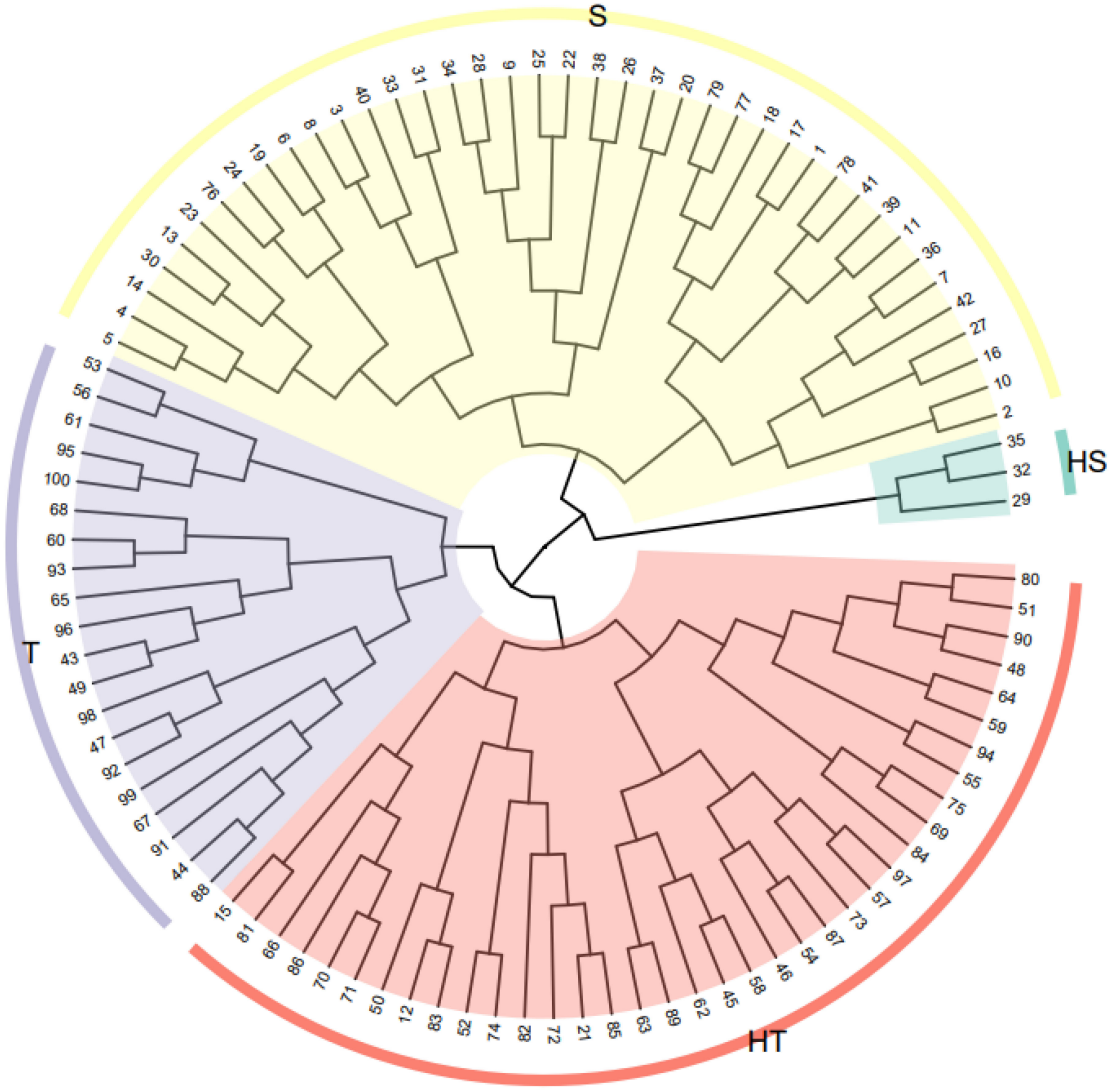
Cluster analysis diagram showing the sorghum genotypes (numbers 1–100 are the ranking of the low-N tolerance of each genotype). Red indicates high low-N-tolerant genotypes (HT), purple indicates low-N tolerant-genotypes (T), yellow indicates low-N-sensitive genotypes (S), and green indicates high-low-N-sensitive genotypes (HS).

A comprehensive index score was obtained using the values Y1, Y2, and Y3 as follows:


(5) Y=0.442Y1+0.246Y2+0.122Y3

where Y is the comprehensive evaluation value of low-N tolerance of sorghum inbred lines under N stress. The higher the Y value, the stronger the resistance to low N stress of sorghum inbred lines; the lower the Y value, the weaker the resistance of sorghum inbred lines to low N stress. This method was used to sort low-N tolerance values in 100 sorghum inbred lines. They were then further clustered by Y values (**Supplementary Table S4**), which were segmented into four categories: high-low-N tolerance, low-N tolerance, low-N sensitivity, and high-low-N sensitivity. The order of the Y values was 1–42, 43–73, 74–97, and 98–100, respectively. The accuracy of genotype selection was determined by cluster analysis using a Euclidean distance of 5 (**Figure 3**). The Y values of the top-ranked genotypes were higher, indicating that the genotypes had a higher tolerance to low-N stress. In contrast, the Y values of the later-ranked genotypes were lower, indicating that they had higher sensitivity to low-N stress. To integrate the phenotypic, micromorphological, and physiological responses of the different low-N-tolerant sorghum genotypes, a low-N-tolerant genotype (398B) and a low-N-sensitive genotype (CS3541) were selected for analysis.

### 3.2 Morphological responses of two sorghum inbred lines to low-N stress

#### 3.2.1 Phenotype

**Figure 4:**
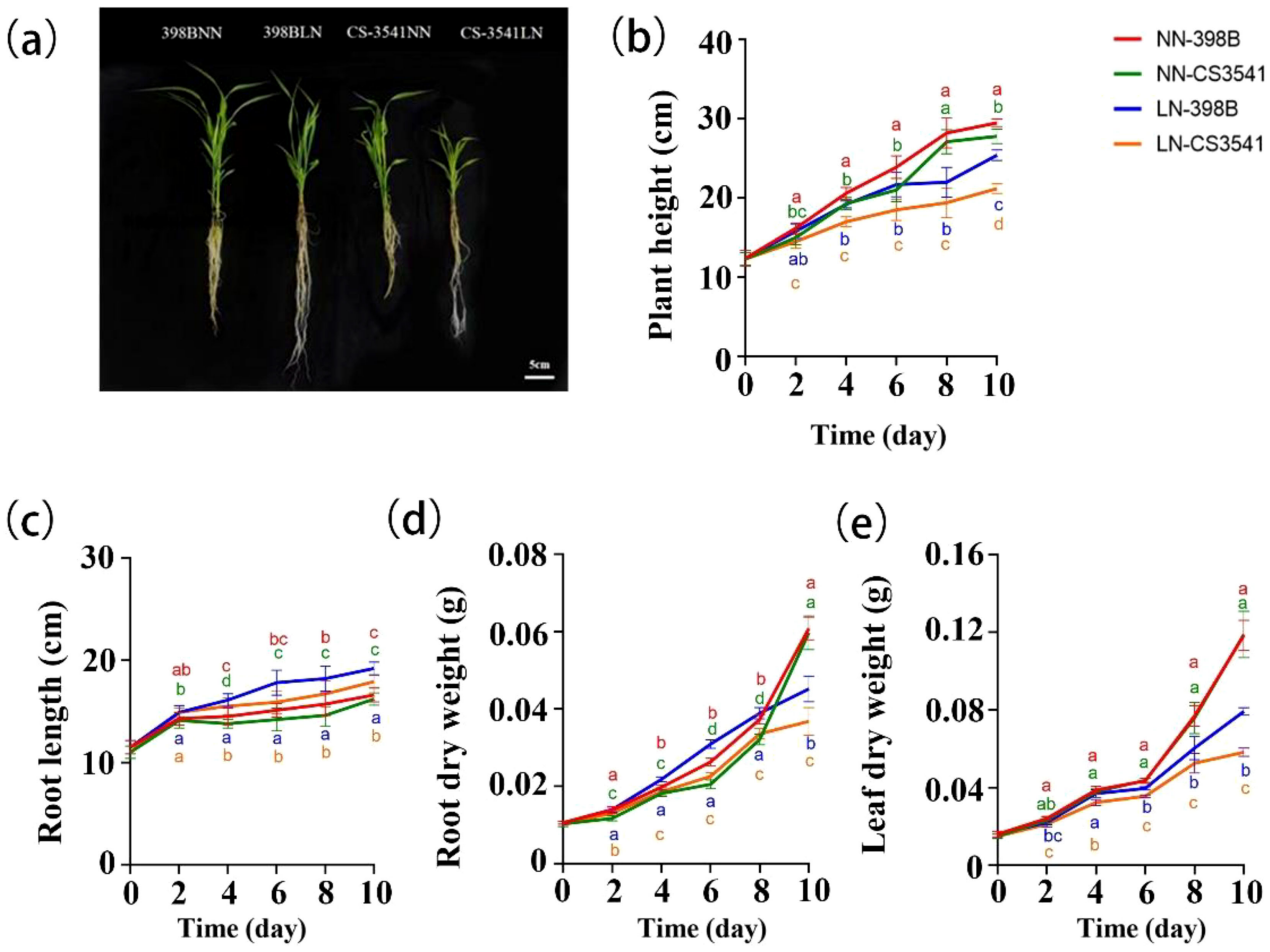
Growth characteristics of the two genotypes after normal-N (NN) and low-N (LN) treatment. Phenotypes of the low-N-tolerant genotype (398B) and low-N-sensitive genotype (CS3541) after 10 days of NN and LN treatments **(A)**. Changes in plant height **(B)**, root length **(C)**, root dry weight **(D)**, and leaf dry weight **(E)** after 0, 2, 4, 6, 8, and 10 days of the NN and LN treatments. Bar, 5 cm. Different letters indicate significant differences (*P <* 0.05).

Sorghum shoot growth was inhibited by low-N levels, whereas root growth was promoted in both sorghum genotypes. However, the degree of inhibition differed between the two genotypes. Apparent symptoms of stress were surveyed for plant height and root length (**Figure 4**). Plant height, shoot dry weight, and root dry weight were significantly reduced under low-N stress. After 10 days of low-N stress, 398B exhibited superior agronomic characteristics, indicating a greater tolerance to low-N stress. The plant height, root dry weight, and leaf dry weight of 398B plants were significantly reduced by 13.89%, 25.66%, and 33.02%, respectively, compared with those of the control. Conversely, CS3541 exhibited reductions of 23.74%, 38.36%, and 50.97%, respectively. Moreover, the plant height of 398B was significantly greater than that of CS3541 after 2, 4, 6, 8, and 10 days of normal-N and low-N treatment, whereas the leaf dry weight of 398B was significantly greater than that of CS3541 after 4, 6, 8, and 10 days of normal-N and low-N treatment.

In contrast, the root length of sorghum was greater under low-N stress in both 398B and CS3541 plants. In comparison with the control treatment, the root lengths of 398B and CS3541 plants were significantly increased by 15.66% and 10.49%, respectively, after 10 days of low-N stress. The root lengths of 398B under low-N stress were 16.14, 17.80, 18.23, and 19.15 cm, which were 4.24%, 11.67%, 9.38%, and 6.85% higher than those of CS3541 after 4, 6, 8, and 10 days of low-N stress, respectively. The root dry weight of 398B was significantly higher than that of CS3541 after 2, 4, 6, 8, and 10 days of normal- and low-N treatment. This phenomenon was observed in the roots of sorghum plants, with the first part of the experiment demonstrating pronounced sensitivity to low-N environments.

#### 3.2.2 Leaf and root microstructures

**Figure 5:**
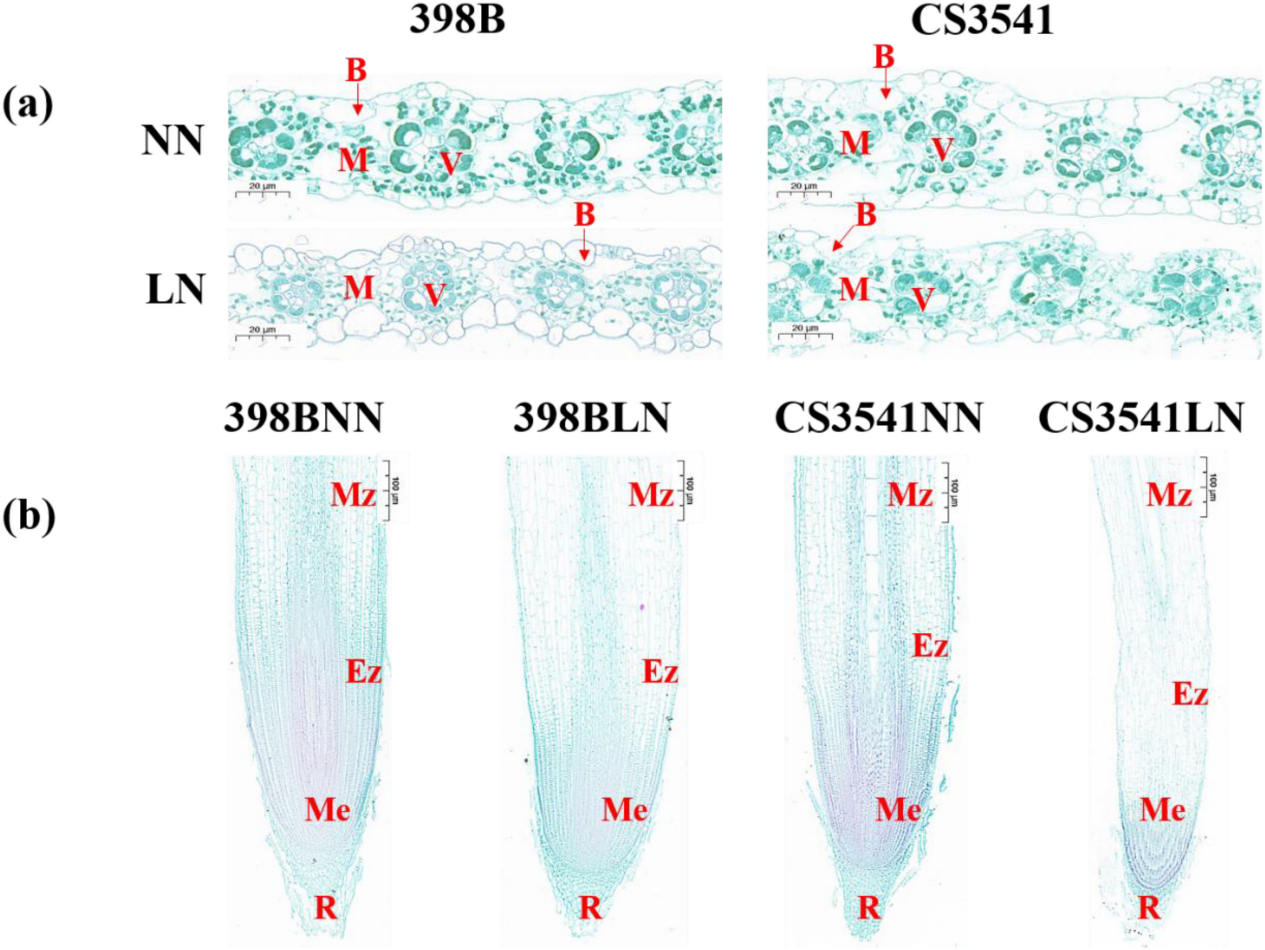
Effects of low-N stress on leaf and root microstructure in sorghum seedlings. **(A)** Changes in leaf microstructure after 10 days of normal-N (NN) and low-N (LN) treatment. ×40 magnification; scale bar, 20 μm. B, bulliform cells; V, vascular bundles; M, mesophyll tissue. **(B)** Changes in root microstructure after 10 days of NN and LN treatment. ×10 magnification; scale bar, 10 μm. R, root cap; Me, meristematic zone; Ez, elongation zone; Mz, maturation zone.

The microstructures of the leaves and roots of 398B and CS3541 were examined and compared (**Figure 5**). Observations of cross-sections of the sorghum leaves revealed the presence of the vascular tissue, the chlorenchyma composed of mesophyll cells, bulliform cells, and the epidermis (**Figure 5A**). The vascular tissue and chlorenchyma are of particular interest in the context of this study. Both genotypes exhibited organized bulliform cells and visible garland structures under normal-N conditions. However, under low-N stress, there was a noticeable degradation of bulliform cells and garland structures in CS3541 leaves, but which were not found in 398B leaves. Leaf thickness, mesophyll thickness, leaf epidermal thickness, vascular bundle cross-sectional area, blade duct area, and blade diameter were also measured (**Supplementary Figures S1, S2**). Low-N stress significantly decreased leaf thickness in CS3541 (29.20%), whereas there was no significant variation in 398B. Under low-N treatment, 398B leaves showed significantly greater leaf thickness and leaf epidermal thickness compared to CS3541, with increases of 21.59% and 128.93%, respectively. Comparing the two genotypes under normal-N treatment, there was a significant reduction in blade duct area and blade diameter in 398B and a similar reduction in the cross-sectional area of the vascular bundle, blade duct area, and blade diameter in CS3541. It was noteworthy that the cross-sectional area of the vascular bundle was generally higher (25.41%) in 398B than in CS3541.

As illustrated in **Figure 5B**, the roots of 398B exhibited denser cells in the meristematic zone and larger cells in the root elongation zone compared with the CS3541 plants. Additionally, when comparing both genotypes under low-N conditions to the control N level, there was a significant increase in the number of cells in the meristematic zone and the length of cells in the elongation zone at the root tips (**Supplementary Figure S3**). The number of cells in the meristematic zone of the root tips were comparable between 398B and CS3541 under control N supply. However, 398B had a higher number of cells in the meristematic zone than CS3541 under low-N condition. Specifically, 398B showed a cell count of 869 in the meristematic zone under low-N stress, which was 43.64% greater than that of CS3541 under the same treatment (**Supplementary Figure S3A**). In terms of cell length, 398B roots measured 345.33 μm under low-N stress, a 61.67% increase compared to 213.60 μm observed in CS3541 under a similar treatment (**Supplementary Figure S3B**). These findings suggest that increased cell division and expansion in the roots may be key adaptive mechanisms that enhance sorghum’s ability to extend root length in response to low-N stress.

### 3.3 Physiological responses of two sorghum inbred lines to low-N stress

#### 3.3.1 Leaf area and photosynthetic parameters

**Figure 6:**
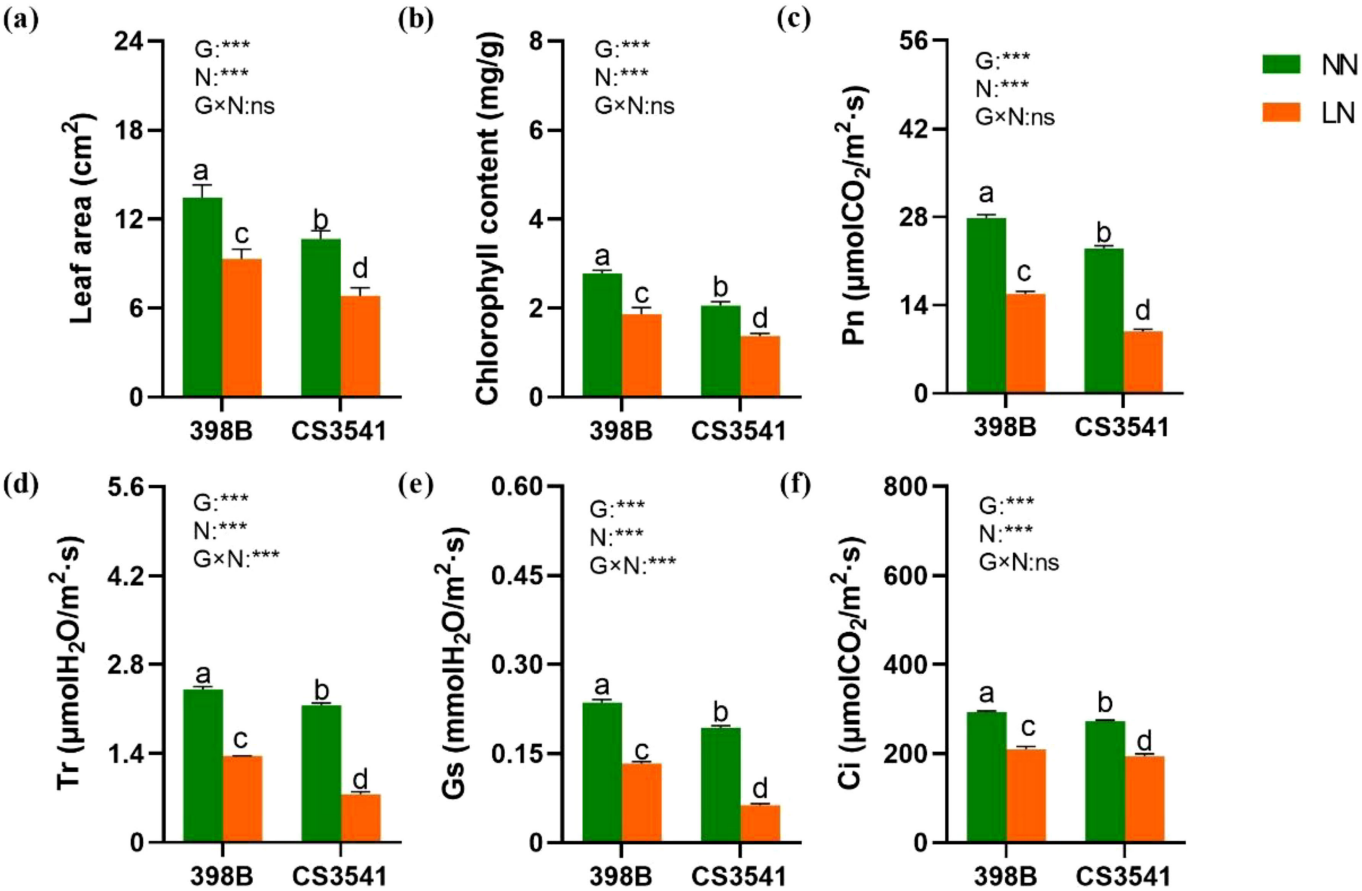
Effects of low-N stress on leaf area and photosynthetic characteristics in sorghum seedlings. Changes in leaf area **(A)**, chlorophyll content **(B)**, Pn **(C)**, Tr **(D)**, Gs **(E)**, and Ci **(F)** after 10 days of normal-N (NN) and low-N (LN) treatment. Pn, net photosynthetic rate; Gs, stomatal conductivity; Tr, transpiration rate; Ci, intercellular CO_2_ concentration. Different lowercase letters indicate significant differences among the different genotypes (*P <* 0.05) under normal-N (NN) and low-N stress (LN). The *P*-values of ANOVA for N treatment, genotypes (G), and their interactions are indicated. ***P < 0.001; ns, no significance.

The effect of low-N stress on leaf area and photosynthetic parameters was more pronounced in CS3541 plants than in 398B plants (**Figure 6**). Low-N stress reduced the leaf area of 398B and CS3541 plants by 30.74% and 36.01%, respectively (**Figure 6A**). Chlorophyll content was decreased by low-N stress, and the degree of this reduction was significantly affected by genotype and N treatment (**Figure 6B**). However, compared with CS3541, the leaf area and chlorophyll content of 398B under low-N treatment were 36.30% and 36.08% higher, respectively. Compared with the normal-N treatment, four parameters (Pn, Gs, Tr, and Ci) in 398B exhibited notable declines of 43.34%, 43.64%, 28.34%, and 43.54%, respectively. Conversely, these leaf photosynthetic parameters in CS3541 were reduced more pronouncedly (57.12%, 67.22%, 28.85%, and 64.77%, respectively; **Figures 6C–F**). In contrast, the Pn, Gs, Tr, and Ci values of 398B exhibited notable increases of 59.92%, 11.70%, 110%, and 8.08%, respectively, compared with those of CS3541 under low-N treatment. As anticipated, the low-N-tolerant sorghum genotype 398B exhibited the most pronounced increase in leaf area and photosynthetic parameters.

The data indicate that low-N levels elicit disparate responses in shoot growth, root morphology, and leaf photosynthesis in 398B and CS3541 plants. This result is likely due to genotypic differences in other metabolic processes, such as N and C metabolism.

#### 3.3.2 N metabolism

**Figure 7:**
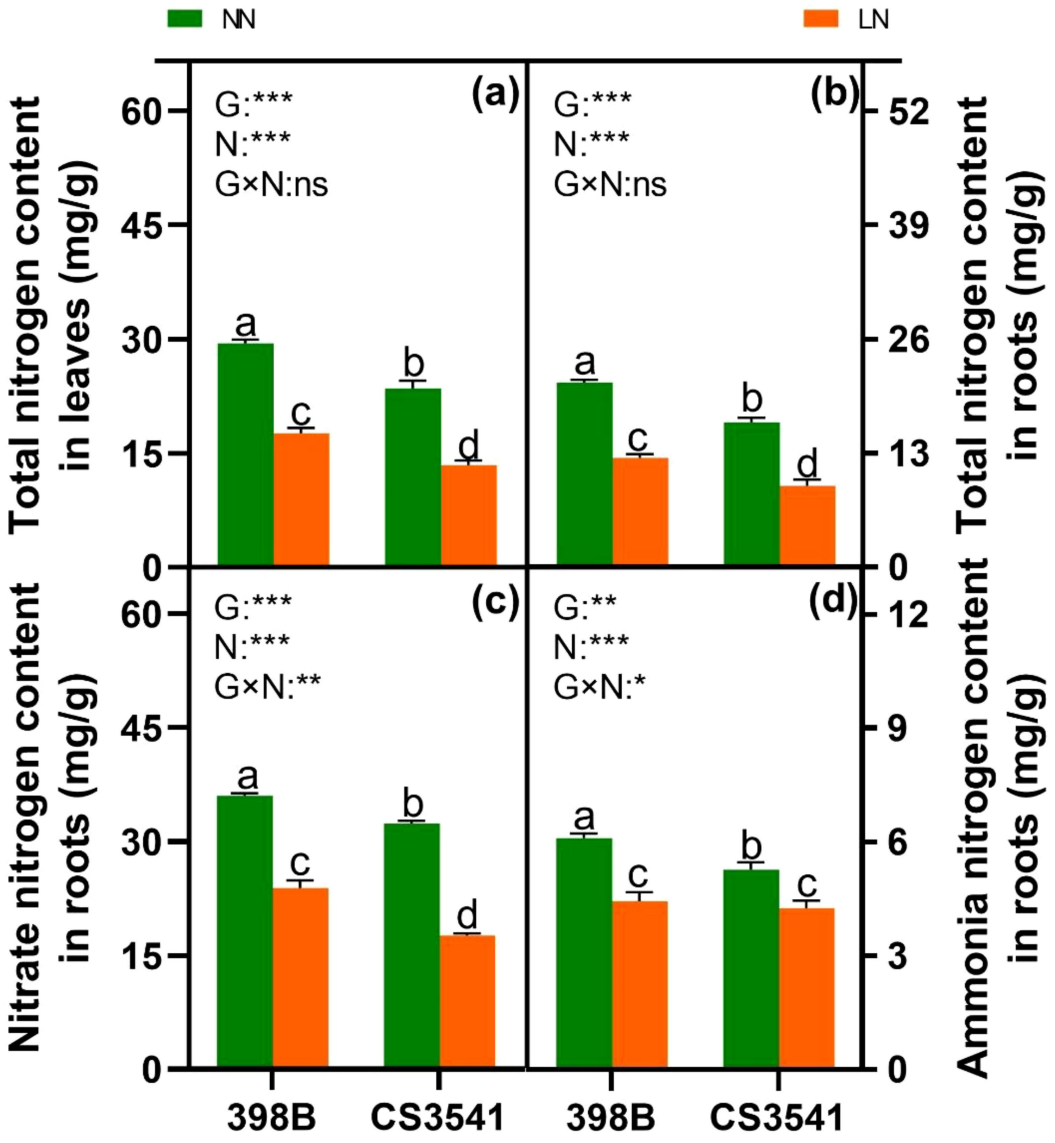
Effects of low-N stress on total N content and NO_3_^-^ and NH_4_^+^ content in sorghum seedlings. Changes in total N content in leaves and roots **(A, B)**; NO_3_^-^ and NH_4_^+^ content in roots **(C, D)** after 10 days of normal-N (NN) and low-N (LN) treatment. Different lowercase letters indicate significant differences among the different genotypes (*P <* 0.05) under normal-N (NN) and low-N stress (LN). The *P*-values of ANOVA for N treatment, genotypes (G), and their interactions are indicated. *P < 0.05; **P < 0.01; ***P < 0.001; ns, no significance.

The total N content in the leaves and roots, respectively, as well as the nitrate and ammonium concentrations in the roots, was quantified to investigate the impact of low-N stress on the N metabolism of sorghum seedling leaves (**Figure 7**). The total N content in the leaves and roots of the 398B plants was higher than that in CS3541 plants under both low- and high-N levels (**Figures 7A, B**). In comparison with normal-N levels, the NO_3_^-^ and NH_4_^+^ content in the roots of both genotypes decreased in response to low-N supply (**Figures 7C, D**). However, the root NH_4_^+^ content was comparable between 398B and CS3541 under low-N stress, whereas NO_3_^-^ content was higher in the roots of 398B than in those of CS3541 under low- and high-N levels.

**Figure 8:**
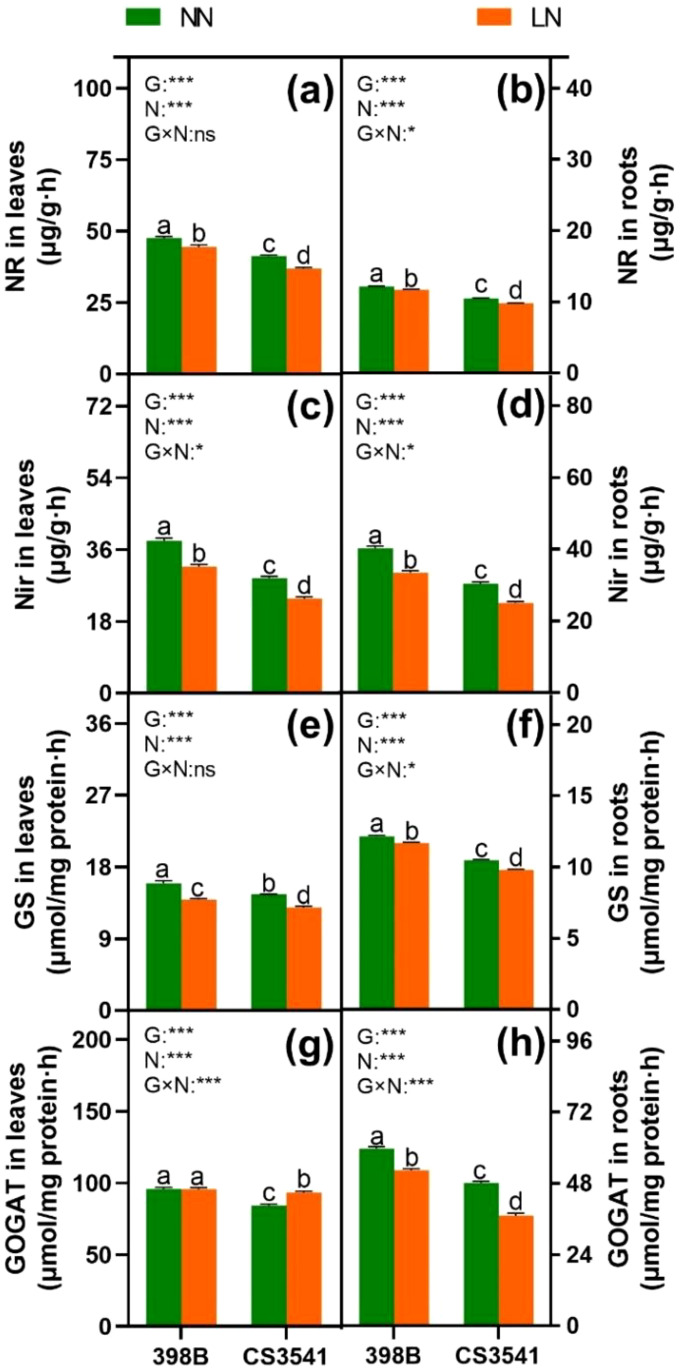
Effects of low-N stress on leaf and root N metabolism enzyme activities in sorghum seedlings. Changes in the NR of leaves and roots **(A, B)**, NiR of leaves and roots **(C, D)**, GS of leaves and roots **(E, F)**, and GOGAT of leaves and roots **(G, H)** after 10 days of normal-N (NN) and low-N (LN) treatment. NR, nitrate reductase; NiR, nitrite reductase; GS, glutamine synthetase; GOGAT, glutamate synthase. Different lowercase letters indicate significant differences among the different genotypes (*P <* 0.05) under normal-N (NN) and low-N stress (LN). The *P*-values of ANOVA for N treatment, genotypes (G), and their interactions are indicated. *P < 0.05; ***P < 0.001; ns, no significance.

After the uptake of nitrate and ammonium, enzymes involved in N metabolism play a pivotal role in N assimilation. Consequently, the current study quantified the activities of N-metabolism enzymes, including NR, nitrite reductase (NiR), GS, and glutamate synthase (GOGAT), which are involved in N assimilation in 398B and CS3541. In the leaves, the activities of NR, NiR, and GS were significantly higher in 398 B than in CS3541, regardless of the N supply (**Figures 8A, C, E**). However, leaf GS activity was comparable between 398B and CS3541 under normal-N conditions but lower in CS3541 than in 398B under low-N supply conditions (**Figure 8G**). Compared with CS3541, the leaf NR, NiR, GS, and GOGAT activities of 398B under low-N treatment were 7.65%, 33.55%, 7.65%, and 2.68%, respectively. Similarly, root NR, NiR, and GS activities were higher in 398B than in CS3541, regardless of the N level (**Figures 8B, D, F, H**). Conversely, the NR, NiR, GS, and GOGAT activities of 398B exhibited notable increases of 17.56%, 4.32%, 3.99%, and 5.92%, respectively, compared with CS3541 under low-N treatment. Furthermore, a significant genotype × N interaction was observed in root NR, NiR, GS, and GOGAT activities, indicating that the root system exhibited a stronger physiological responsiveness to low-N availability.

#### 3.3.3 Leaf amino acid content

**Figure 9:**
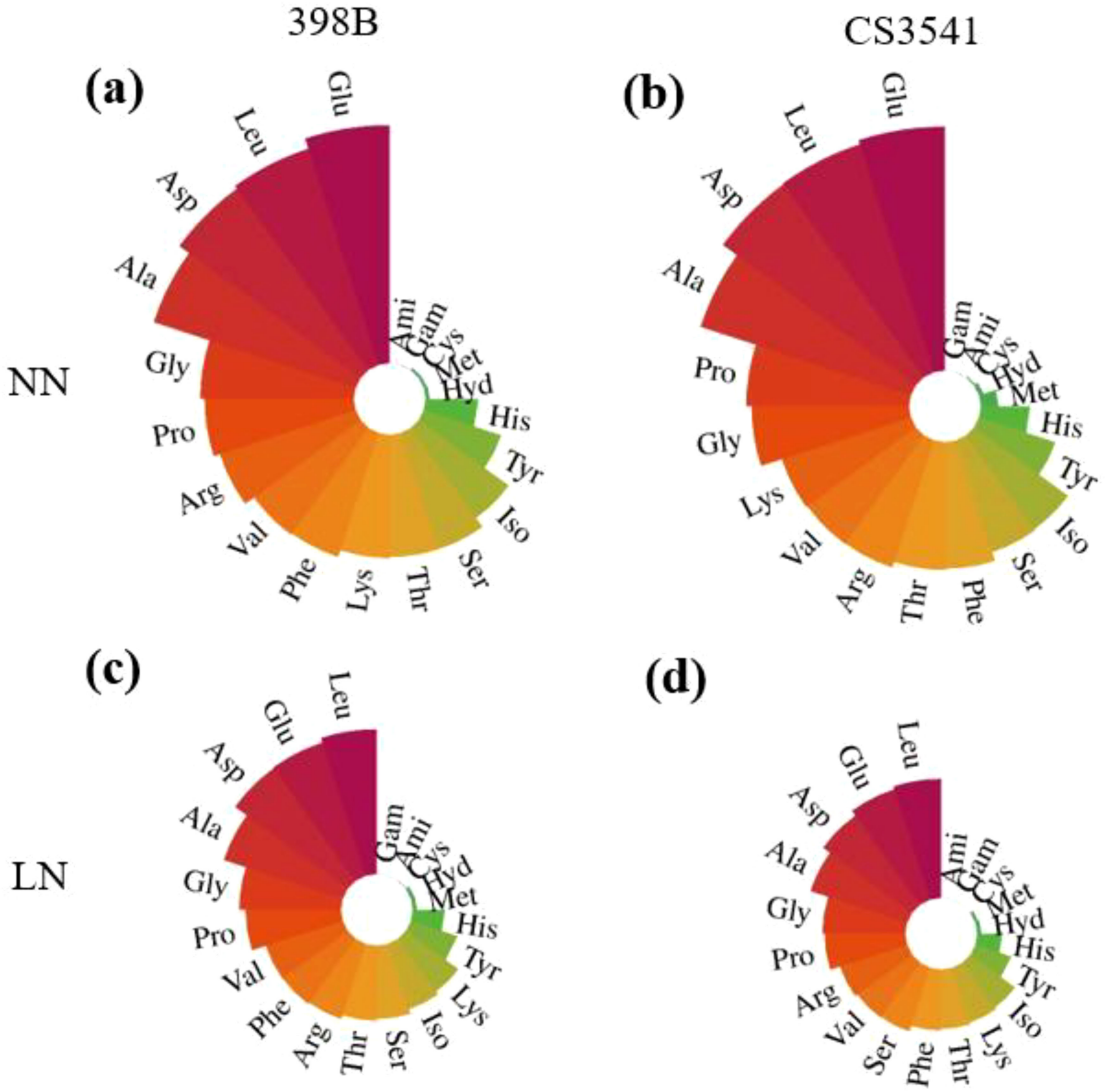
Effects of low-N stress on leaf amino acid content in sorghum seedlings. Changes in amino acid content of 398B leaves after 10 days of normal-N (NN) treatment **(A)**, CS3541 leaves after 10 days of NN treatment **(B)**, 398B leaves after 10 days of low-N (LN) treatment **(C)**, and CS3541 leaves after 10 days of LN treatment **(D)**. Amino acids: His, histidine; Hyd, 4-hydroxy-L-proline; Arg, arginine; Ser, serine; Gly, glycine; Asp, aspartic acid; Glu, glutamic acid; Thr, threonine; Ala, alanine; Gam, gamma-aminobutyric acid; Pro, proline; Ami, D-2-aminobutyric acid; Lys, lysine; Cys, cystine; Met, methionine; Tyr, tyrosine; Val, valine; Iso, isoleucine; Leu, leucine; Phe, phenylalanine.

To investigate the N metabolic products involved in N assimilation, the contents of free amino acids, including arginine, glutamic acid, proline, histidine, 4-hydroxy-L-proline, serine, glycine, aspartic acid, threonine, alanine, lysine, cysteine, methionine, tyrosine, valine, isoleucine, leucine, and phenylalanine, were quantified (**Figure 9**). Except for 4-hydroxy-L-proline, the contents of the remaining free amino acids were higher in 398B leaves than in CS3541 leaves. Histidine, glycine, threonine, cysteine, tyrosine, isoleucine, leucine, and phenylalanine contents were significantly higher in 398B leaves than in CS3541 leaves under low-N supply conditions. The histidine, glycine, threonine, cysteine, tyrosine, isoleucine, leucine, and phenylalanine content in 398B decreased by 1.63-, 1.51-, 1.63-, 3.69-, 1.67-, 1.62-, 1.57-, and 1.62-fold for 398B compared with the normal-N treatment, whereas they were 1.95-, 1.89-, 2.15-, 4.23-, 2.12-, 2.09-, 2.01-, and 2.07-fold, respectively, in the CS3541 group. These findings indicate that 398B may exhibit greater amino acid biosynthesis capacity under low-N conditions.

#### 3.3.4 C metabolism

**Figure 10:**
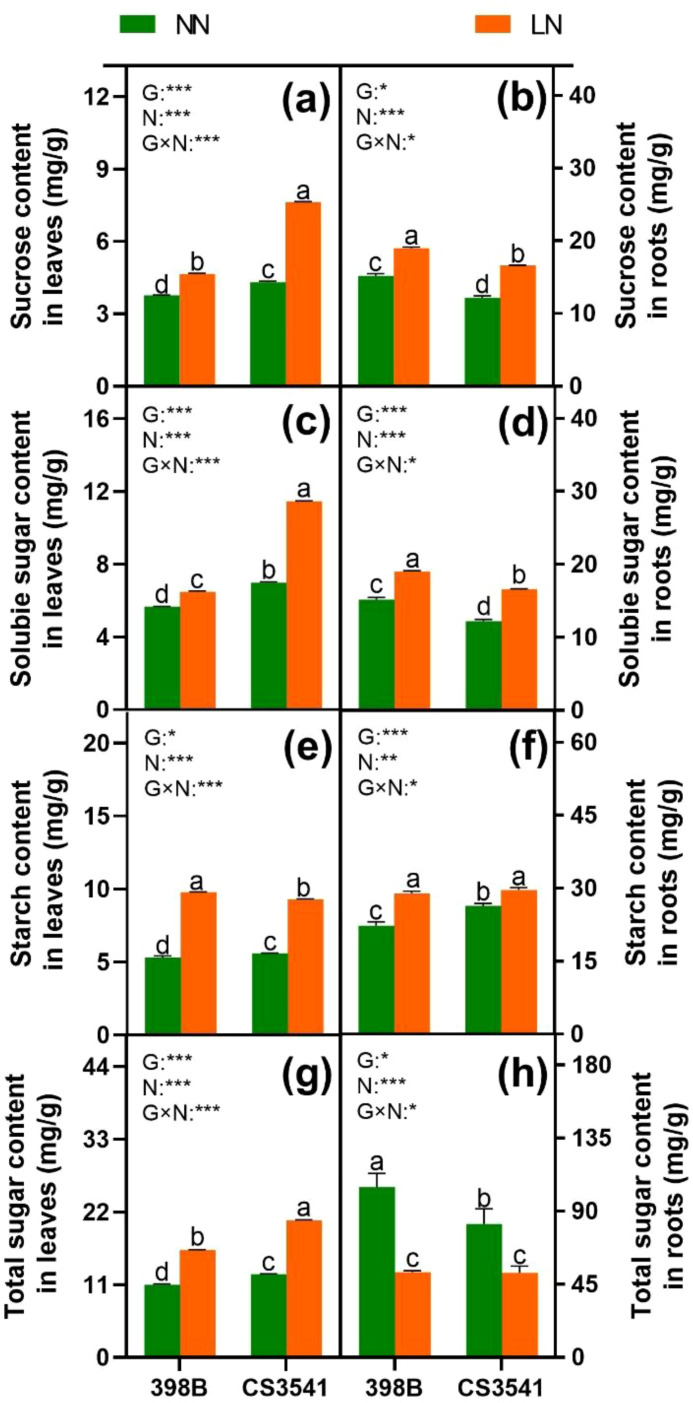
Effects of low-N stress on leaf and root sucrose, soluble sugar, starch, and total sugar in sorghum seedlings. Changes in sucrose content **(A, B)**, soluble sugar content **(C, D)**, starch content **(E, F)**, and total sugar content **(G, H)** of leaves and roots, respectively, after 10 days of normal-N (NN) and low-N (LN) treatments. Different lowercase letters indicate significant differences among the different genotypes (*P <* 0.05) under normal-N (NN) and low-N stress (LN). The *P*-values of ANOVA for N treatment, genotypes (G), and their interactions are indicated. *P < 0.05; **P < 0.01; ***P < 0.001.

**Figure 11:**
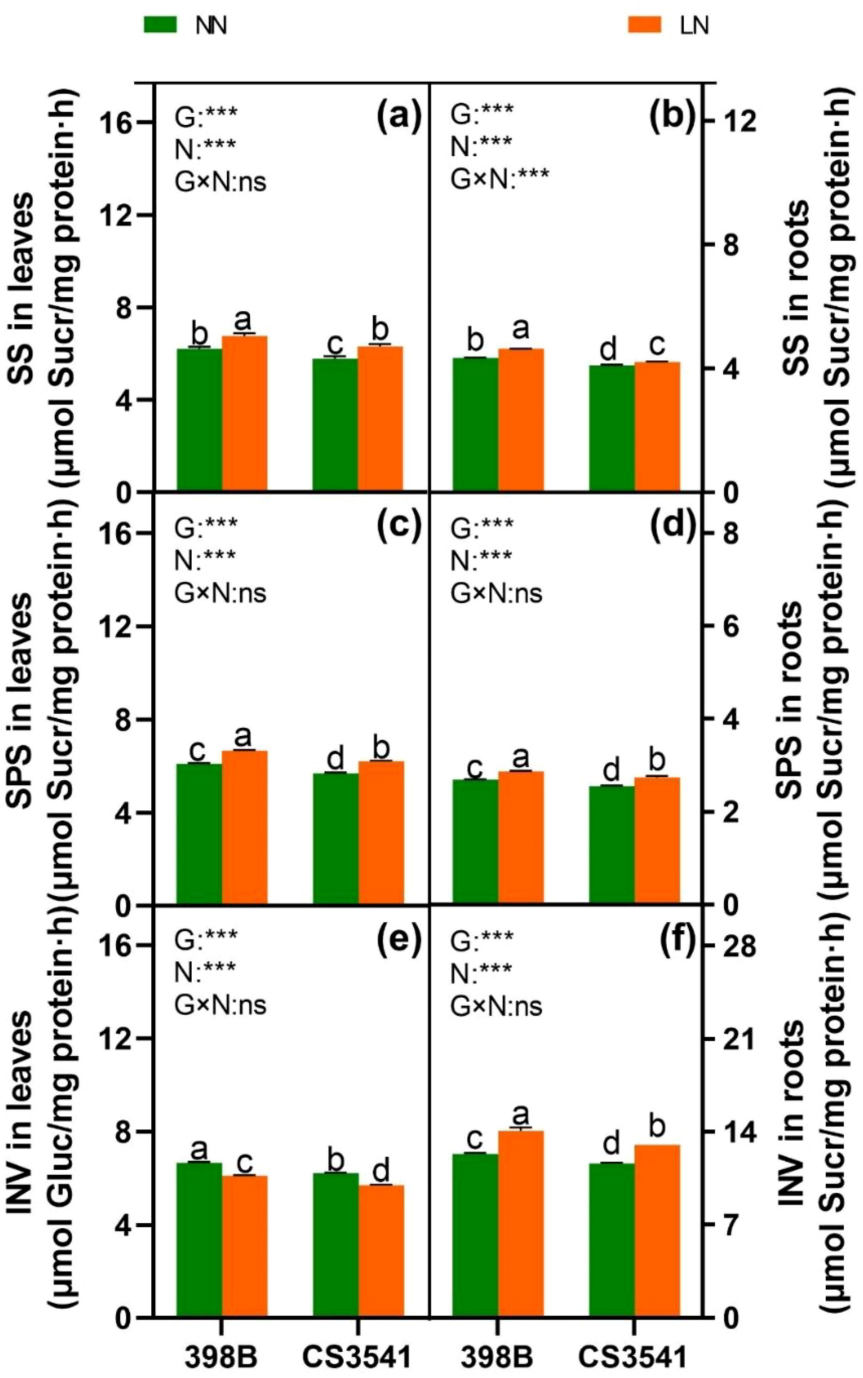
Effects of low-N stress on leaf and root C metabolism enzyme activities in sorghum seedlings. Changes in SS **(A, B)**, SPS **(C, D)**, and INV **(E, F)** in leaves and roots, respectively, after 10 days of normal-N (NN) and low-N (LN) treatments. SPS, sucrose phosphate synthase; SS, sucrose synthase; INV, invertase. Different lowercase letters indicate significant differences among the different genotypes (*P <* 0.05) under normal-N (NN) and low-N stress (LN). The *P*-values of ANOVA for N treatment, genotypes (G), and their interactions are indicated. ***P < 0.001; ns, no significance.

To determine the capacity for C metabolism, the assimilation of C skeletons was assessed (**Figures 10**, **11**). The results demonstrated that sucrose, starch, and soluble sugar levels were higher in low-N plants than in normal-N plants of both genotypes, except for total sugar content in the roots. The sucrose and soluble sugar contents were significantly higher in CS3541 than in 398B, in contrast to the roots. Leaf total sugar content was higher in CS3541 than in 398B, regardless of the N supply. Nevertheless, no significant differences in total sugar content were observed between 398B and CS3541 in the roots of N-starved plants.

The activity values of SS, SPS, and INV were also analyzed. Regardless of the N supply, SS and SPS were greater in the leaves and roots of 398B than in CS3541. In contrast, leaf INV activity was significantly reduced, whereas root INV activity was significantly increased in both genotypes in response to low-N levels. However, it was significantly higher in 398B than in CS3541 in the leaves of N-starved plants and higher in 398B than in CS3541 in the roots of N-replete plants. Furthermore, a significant genotype-by-N interaction was observed in leaf and root SS, SPS, and INV activities, indicating complex patterns in the responses of N nutrient elements and C-bearing compounds to N supply levels.

## 4 Discussion

Sorghum is a C_4_ crop with high N use efficiency. However, 8%–15% of its production costs are attributed to fertilizer, underscoring the importance of sufficient soil nutrients, particularly N, to achieve optimal yield (Olson et al., 2013). To address this issue, a comprehensive and systematic evaluation of low-N tolerance in sorghum is required to facilitate its strategic development and deployment as a pioneer crop.

### 4.1 Evaluation of low-N stress tolerance in sorghum at seedling stages

Crops often exhibit morphological and physiological responses to environmental stress, with the seedling stage being the primary and most sensitive stage of development. Consequently, seedling growth parameters must be incorporated into low-N tolerance evaluations. Evaluating seedling growth in low-N environments is essential to elucidate the low-N tolerance of sorghum. However, there is currently a paucity of systematic studies on low-N tolerance in sorghum. Zhang et al. (2017) compared nine genotypes of *Fagopyrum tararicum* and identified relative plant height, stem diameter, root/shoot ratio, leaf area, and chlorophyll concentration as indicators of low-N tolerance. This study further demonstrated that these indicators can be used to evaluate the tolerance of various genotypes, revealing similar patterns of morphological changes under low-N stress, although the extent of these changes varied among genotypes. PCA, Pearson’s correlation analysis, and Y value comprehensive analysis were used to evaluate the tolerance of 100 sorghum genotypes under N stress. The identified sorghum indicators were classified into three factors, with the maximum eigenvector load corresponding to plant height, shoot dry weight, and root dry weight. Ultimately, this systematic approach allowed the selection of 100 sorghum genotypes for a detailed evaluation of low-N tolerance during the seedling stage. The genotypes were then categorized into four groups according to tolerance levels. Identification and validation of low-N tolerance indicators are vital for breeders and will help improve available genetic resources. Furthermore, future studies should focus on employing dimensionality reduction techniques, simplifying complex traits, and applying visualization methods to provide a more detailed reflection of low-N tolerance across various sorghum genotypes.

### 4.2 Morphological and photosynthetic responses to low-N stress in sorghum seedlings

Plants can adapt to diverse environmental stimuli by modifying their fundamental root structures—for example, plants can adjust their root architecture to enhance N acquisition under N-deficient conditions, thereby adapting to an N-deficient environment. The “steep, cheap, and deep” ideotype for maize (*Zea mays*) exemplifies this concept, proposing that root phenotypes optimize N capture under limited N resource availability (Gao et al., 2015). The results of our study indicate that the root morphologies of the two sorghum genotypes exhibit distinct responses to N-deficient conditions. Specifically, genotype 398B had a longer root length and greater root dry weight than CS3541. This suggests that a plastic root system can enhance low-N tolerance in sorghum. Furthermore, faster root growth rates are conducive to plant growth (Yuan et al., 2022). Similar responses to N starvation were observed in terms of plant height and shoot dry weight in 398B and CS3541 plants under low-N conditions. However, 398B plants exhibited greater plant height and shoot dry weight, indicating higher N assimilation efficiency during growth.

The architecture of the root system, which is the physical configuration of the roots, governs their distribution within the soil over time and space, making it a primary determinant of N capture, especially in low-N environments. Anatomical phenotypes that reduce the carbon and nutrient requirements of root growth and maintenance should consequently improve the acquisition of soil N resource (Sun et al., 2018; Lynch et al., 2021). Lynch et al. (2023) indicated that an elongation of root cortical cells can ameliorate N capture by accelerating the root elongation rate, thereby improving the efficiency of N capture and utilization. This is corroborated by our study that N starvation triggered an increase in cortical cell length in the root elongation zone, which, in turn, altered the anatomical structure to support root elongation under low-N conditions. Root growth is characterized by its indeterminate nature, marked by the continual succession of cell division, regulated cell expansion, and differentiation within the meristem and adjacent root regions. Root morphogenesis is controlled by the interplay of cell division and expansion regulation (Strader et al., 2010). In this study, it was observed that the meristematic zone of the roots in both sorghum genotypes exhibited a denser packing of cells compared to those grown under N-sufficient conditions. It was particularly noteworthy that 398B had a significantly higher number of cells in the meristematic zone of the root tips compared to CS3541 under low-N stress. As soil hardness typically escalates with depth, hindering the capture of N from deep soil, longer roots that can penetrate hard soil may also improve the capture of N from deep regions. Both cell division and expansion were pivotal phenotypic traits that merit further investigation as an avenue to improve N capture and use efficiency in sorghum, especially in response to N deficiency.

The leaf blade is specialized to capture light and utilize the energy for photosynthetic carbon assimilation while also being capable of limiting N content, surface area, and resistance for gas exchange (Wakayama et al., 2006). The specialized anatomy requires the development of two distinct chloroplast-containing cell types within the leaf: the mesophyll cells, which constitute the majority of cells within the leaf and are predominantly located between the parallel vascular strands, and the bundle sheath cells, which encircle the vascular tissue, forming a column of cells that extend along the length of each vascular strand (Smillie et al., 2012). Under low-N stress, we observed a degradation of bulliform cells and garland structures in CS3541 leaves, which was not apparent in the leaves of 398B. Furthermore, there was no significant variation in leaf thickness and the cross-sectional area of vascular bundles in 398B. However, a greater leaf epidermal thickness and a larger cross-sectional area of vascular bundle were observed in 398B leaves than in CS3541 leaves under low-N stress. This suggests that 398B accumulates biomass in leaves and promotes robust leaf growth and development by maintaining a stable internal leaf structure. The photosynthetic parameters, including photosynthetic rate (Pn), transpiration rate (Tr), intercellular CO₂ concentration (Ci), and stomatal conductance (Gs), serve as internal markers of photosynthetic metabolism in plants (Liu et al., 2022b; Qin et al., 2021). Therefore, it can be postulated that improvements in these gas exchange parameters under stress conditions could bolster plant stress resistance. Zhao et al. (2005) found that N deficiency significantly reduced the leaf chlorophyll concentration, the rate of leaf net photosynthesis, and biomass production in sorghum. In this study, Pn, Tr, Ci, and Gs were significantly reduced in 398B and CS3541 plants under N stress. However, the decrease was more pronounced in CS3541 than in 398B, suggesting that the N-efficient genotype has a higher capacity for C assimilation than the N-inefficient genotype, as discussed further below.

### 4.3 C and N metabolism responses to low-N in sorghum seedlings

Improvements in plant growth in the external environment are often correlated with C and N metabolism (Woo et al., 2004; Meng et al., 2018). In particular, the longer roots in 398B exhibited significantly higher total N and NO_3_^-^ content under low-N conditions than the CS3541 roots. This increase is likely associated with NRT function (Hawkins and Robbins, 2010). NR is a pivotal enzyme that regulates the rate-limiting step of the NO_3_^-^ assimilation pathway. Subsequently, nitrate is converted to ammonium, which serves as a precursor for the synthesis of various amino acids, including glutamate, arginine, proline, and 4-hydroxyproline, via the GS/GOGAT cycle (Xu et al., 2012). The 398B plants were found to have higher NR, NiR, GS, and GOGAT activity levels in their leaves and roots than the CS3541 plants during N starvation. This indicates that 398B may have a greater capacity for amino acid biosynthesis under low-N conditions. This was further supported by the higher concentrations of most amino acids in 398B than in CS3541 under low-N conditions. Consequently, greater N assimilation capacities may enhance low-N tolerance in sorghum.

Glutamic acid (Glu), the primary amino acid produced by the GS/GOGAT cycle, serves as an amino group donor for the synthesis of numerous other amino acids (Forde and Lea, 2007). This study observed a significant reduction in the concentration of glutamic acid (Glu) in sorghum leaves under low-N conditions. Interestingly, no difference in the Glu concentration was observed between the two genotypes under low-N conditions, indicating that the biosynthesis of various amino acids may also be influenced by the scarcity of amino group donors. However, a considerable number of essential amino acids, including histidine, glycine, threonine, cysteine, tyrosine, isoleucine, leucine, and phenylalanine, were found to be significantly higher in 398B plants than in CS3541 plants under low-N stress. This robust individual response to low-N conditions, particularly for leucine, has also been observed in tomato leaves (Urbanczyk-Wochniak and Fernie, 2005). In the face of nutrient limitation, amino acids in leaves are recycled and allocated to young leaves or sink tissues for the synthesis of specific proteins, and this process occurs frequently (Watanabe et al., 2013; Hildebrandt et al., 2015)—for instance, the concentrations of threonine, isoleucine, and leucine were markedly diminished under conditions of low-N availability, which is likely due to the limited availability of C for N assimilation. Furthermore, the levels of glycolysis-derived phenylalanine and tyrosine also significantly decreased under low-N conditions, consistent with the findings of Schlüter et al. (2012). This evidence underscores that N deficiency is accompanied by significant alterations in different amino acids, indicating that amino acids play an important role in the adaptation of source-leaf metabolism to N deprivation and the export of N resources to sink organs.

Photosynthesis and C metabolism are well recognized as primary sources of energy, and the C skeleton is essential for plant growth and N assimilation (Du et al., 2023). Our findings indicate that sucrose, soluble sugar, and total sugar levels were significantly lower in 398B leaves than in CS3541 leaves regardless of N supply. Conversely, the roots exhibit the opposite trend. Consequently, it is postulated that C assimilation in 398B leaves is more efficient than that in CS3541 leaves. Furthermore, under low-N conditions, there was a significant increase in the activities of SS and SPS, whereas INV activity was induced in leaves. It is conceivable that C metabolism may be reprogrammed to favor C storage as starch or sucrose rather than exporting it to achieve a balance between C and N (Lu et al., 2020). In contrast, the roots of both 398B and CS3541 showed lower soluble sugar and total sugar contents but higher sucrose and starch contents under low-N levels. It was speculated that root growth retardation may be due to changes in sink demand, allowing C accumulation under N stress. This occurred despite a significant increase in the activities of SS, SPS, and INV in the roots. Notably, the sucrose content and activities of SS and SPS were greater in 398B roots than in CS3541 roots regardless of N supply. This is consistent with previous studies (Gelli et al., 2014; Bollam et al., 2021). These results indicate that the C/N balance, which is crucial to maintain optimal plant growth and respond to N limitations, is modulated differently in the two sorghum genotypes with contrasting tolerances to low-N. Understanding these genotype-specific responses is vital to develop strategies to enhance crop productivity in N-deficient environments.

## 5 Conclusion

**Figure 12:**
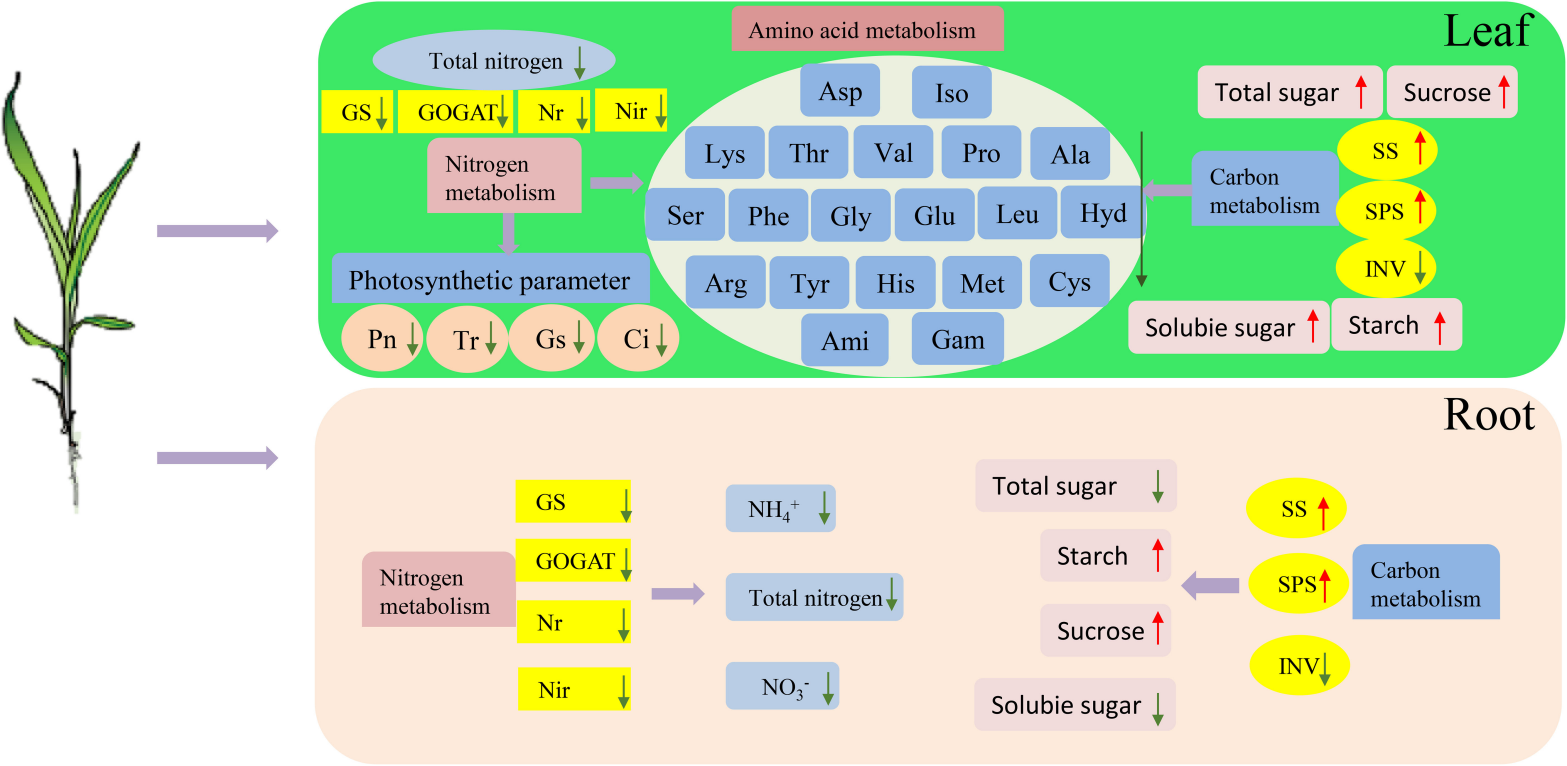
Effects of low-N stress on C and N metabolism in sorghum seedlings. The upward red and downward green arrows represent the increases and decreases, respectively. GS, glutamine synthetase; GOGAT, glutamate synthase; NR, nitrate reductase; NiR, nitrite reductase; SPS, sucrose phosphate synthase; SS, sucrose synthase; INV, invertase; Pn, net photosynthetic rate; Gs, stomatal conductivity; Tr, transpiration rate; Ci, intercellular CO_2_ concentration; Ala, alanine; Ami, D-2-aminobutyric acid; Arg, arginine; Asp, aspartic acid; Cys, cystine; Gam, gamma-aminobutyric acid; Glu, glutamic acid; Gly, glycine; His, histidine; Hyd, 4-hydroxy-L-proline; Iso, isoleucine; Leu, leucine; Lys, lysine; Met, methionine; Phe, phenylalanine; Pro, proline; Ser, serine; Thr, threonine; Tyr, tyrosine; Val, valine.

This study presents a comprehensive evaluation and categorization of the tolerance of 100 sorghum genotypes to low-N stress. The comparative analyses included Pearson’s correlation, PCA, and cluster analysis. Root/shoot ratio, root fresh and dry weight, and root length were the key factors assessed because they are critical in determining low-N tolerance. Low-N-tolerant genotypes exhibited enhanced plant growth and increased leaf photosynthetic capacities. They also showed augmented enzyme activities related to N and C metabolism in both leaves and roots compared with low-N-sensitive genotypes under limited N supply (**Figure 12**). Notably, the low-N-tolerant genotypes exhibited greater amino acid concentrations in their metabolic pool following N deprivation. This robust growth and physiological metabolism strategy may serve as a pivotal mechanism to elucidate the phenomenon of genotypes that exhibit heightened low-N tolerance levels compared with low-N sensitive genotypes.

## Data Availability

The original contributions presented in the study are included in the article/**Supplementary Material**. Further inquiries can be directed to the corresponding author.
