# Supplementary material for: Screening key sorghum germplasms for low-nitrogen tolerance at the seedling stage and identifying from the carbon and nitrogen metabolism
Source: Front Plant Sci. 2024 Sep 12;15:1340509. doi: 10.3389/fpls.2024.1340509 (PMC11424420; doi:10.3389/fpls.2024.1340509)
Supplement: Supplementary file 1 [file DataSheet1.docx]

**
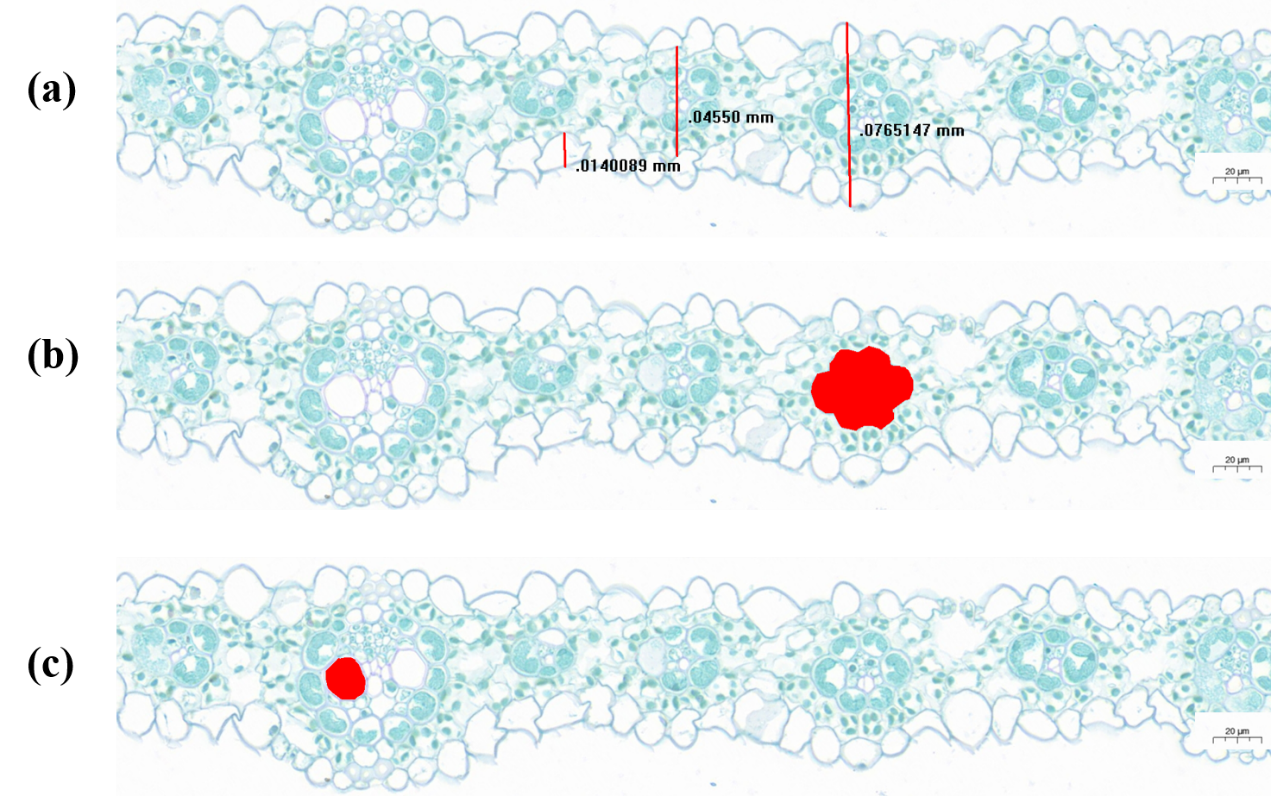
**

**Supplementary Figure 1** Diameter in leaf bulliform cells, the shortest vertical line; diameter in vascular bundle, the second vertical line; leaf epidermal thickness, the longest vertical line **(a)**; cross-sectional area of vascular bundle **(b);** blade duct area **(c)**.

**
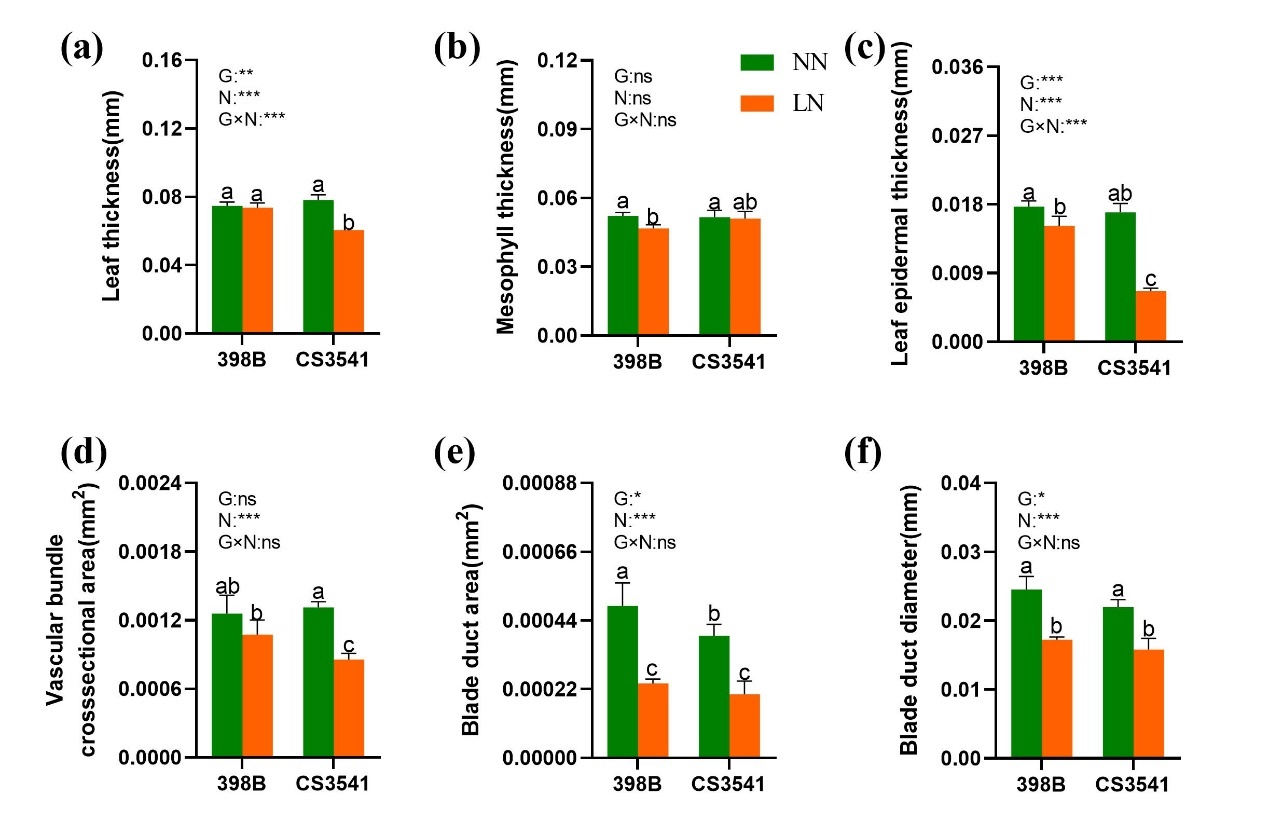
**

**Supplementary Figures 2** Changes in cross-section of leaf thickness **(a)**; leaf mesophyll thickness **(b)**; leaf epidermal thickness **(c)**; cross-sectional area of vascular bundle **(d)**; blade duct area **(e)**; and blade diameter **(f)** after 10 days of normal-N (NN) and low-N (LN) treatments. Different lowercase letters indicate significant differences among the different genotypes (*P* < 0.05) under normal-N (NN) and low-N stress (LN). *P*-values of the ANOVA for N treatment, genotypes (G), and their interactions are indicated. * *P* < 0.05; ** *P* < 0.01; *** *P* < 0.001; ns, no significance.

**
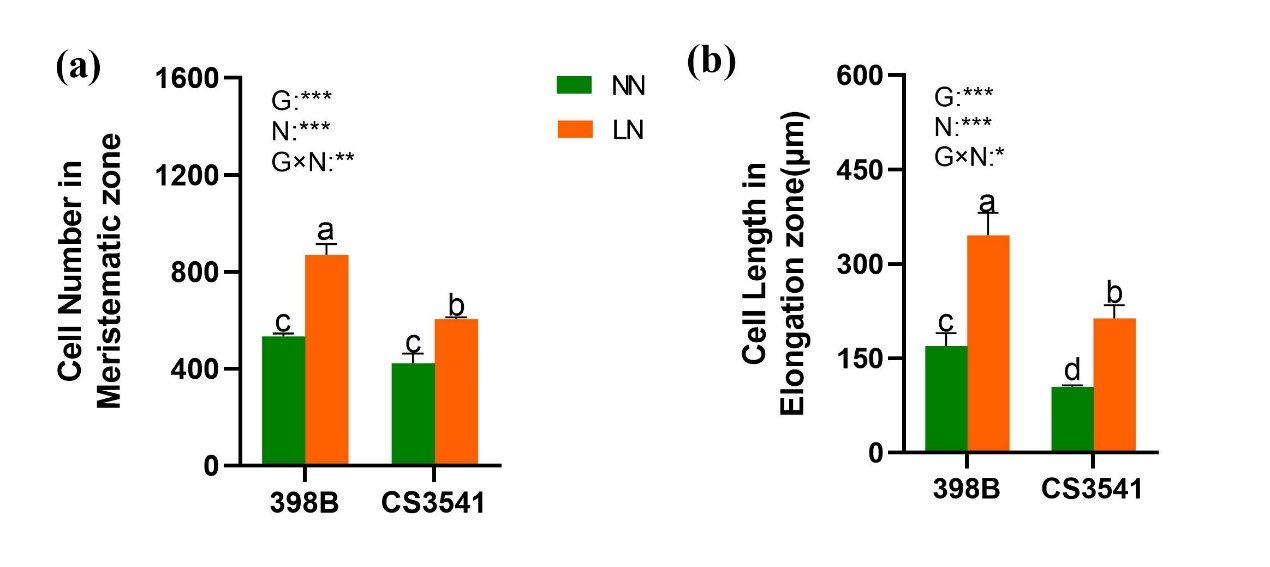
**

**Supplementary Figures 3** Changes in cell number meristematic zone **(a)**; cell length in elongation zone **(b)** after 10 days of normal-N (NN) and low-N (LN) treatments. Different lowercase letters indicate significant differences among the different genotypes (*P* < 0.05) under normal-N (NN) and low-N stress (LN). *P*-values of the ANOVA for N treatment, genotypes (G), and their interactions are indicated. * *P* < 0.05; ** *P* < 0.01; *** *P* < 0.001; ns, no significance.

**Table S1** Name and number of tested sorghum inbred line

| Number | Germplasm name | Number | Germplasm name | Number | Germplasm name |
| --- | --- | --- | --- | --- | --- |
| 1 | MW sorghum | 35 | CS3541 | 69 | 5933 |
| 2 | 20121729 | 36 | NWT sorghum | 70 | JiR105 |
| 3 | LMZQJ sorghum | 37 | Zhe17B | 71 | I1H08313 |
| 4 | HLPFBYsorghum | 38 | HeNong16 | 72 | Nan133 |
| 5 | 7S1450 | 39 | 299 | 73 | 2316B |
| 6 | Sweet sorghum | 40 | ICS11758 | 74 | IS-18947 |
| 7 | PDX sorghum | 41 | IH080313 | 75 | IS10654B |
| 8 | DongYa sorghum | 42 | 7313 | 76 | IS-22204 |
| 9 | Sweet sorghum | 43 | YTB sorghum | 77 | LR116 |
| 10 | GDQ sorghum | 44 | HM sorghum | 78 | T180 |
| 11 | MR-868 | 45 | Tie208 | 79 | P-932127 |
| 12 | ICSV390 | 46 | Zhe13B | 80 | QL33B |
| 13 | 20121733 | 47 | Tie157 | 81 | DQB sorghum |
| 14 | PTZ sorghum | 48 | HaHui75 | 82 | SCS sorghum |
| 15 | ICSV233 | 49 | SC170-14 | 83 | 9198 |
| 16 | ICSV330 | 50 | Sc120-6 | 84 | HBX sorghum |
| 17 | Sweet sorghum | 51 | Sc 170 | 85 | Ji2731B |
| 18 | LNB sorghum | 52 | RANCHER | 86 | EXP23N |
| 19 | ICSV219 | 53 | Zhe23B | 87 | 115B |
| 20 | BDT sorghum | 54 | P-955011 | 88 | M-60251 |
| 21 | M-60035B | 55 | Sg2354 | 89 | 314B |
| 22 | Chi6B | 56 | SC599-11E | 90 | 10164 |
| 23 | HLS sorghum | 57 | JinLiang1 | 91 | 10036 |
| 24 | ENX sorghum | 58 | 0665R | 92 | 10124 |
| 25 | JH13 | 59 | Suda×St11 | 93 | 10144 |
| 26 | IH08031 | 60 | P-932149 | 94 | 10106 |
| 27 | DBL sorghum | 61 | 398B | 95 | 10032 |
| 28 | Black sorghum | 62 | 866R | 96 | 0664R |
| 29 | 7616-533 | 63 | 77CS290-2 | 97 | 0662R |
| 30 | QianGao8 | 64 | HaHui144 | 98 | JinZa18B |
| 31 | HKF sorghum | 65 | ChiHui428 | 99 | TAM428B |
| 32 | MN-4138 | 66 | 0-30 | 100 | TX622B |
| 33 | M-60169 | 67 | I1H08031 |  |  |
| 34 | IH08055 | 68 | I1H08055 |  |  |

**Table S2** Coefficient of tolerance of sorghum growth characteristics to low-N stress at seedling stage

| Traits | Max (%) | Min (%) | Average (%) | Standard (%) | CV (%) |
| --- | --- | --- | --- | --- | --- |
| Plant height | 1.10 | 0.51 | 0.83 | 0.16 | 0.19 |
| Shoot fresh weight | 1.17 | 0.35 | 0.71 | 0.21 | 0.29 |
| Shoot dry weight | 1.18 | 0.43 | 0.81 | 0.20 | 0.24 |
| Root length | 2.03 | 0.57 | 1.17 | 0.28 | 0.24 |
| Root fresh weight | 1.70 | 0.46 | 1.00 | 0.29 | 0.29 |
| Root dry weight | 1.91 | 0.45 | 1.11 | 0.29 | 0.27 |
| SPAD | 1.32 | 0.53 | 0.86 | 0.18 | 0.21 |
| Root N content | 0.98 | 0.05 | 0.57 | 0.19 | 0.34 |
| Shoot N content | 0.97 | 0.28 | 0.61 | 0.17 | 0.28 |
| Root/Shoot | 2.67 | 0.59 | 1.42 | 0.44 | 0.31 |

Max: maximum; Min: minimum; CV: coefficient of variation

**Table S3 Factor score coefficient matrix**

| Factor score coefficient matrix | 1 | 2 | 3 |
| --- | --- | --- | --- |
| Plant height | 0.563 | 0.652 | 0.26 |
| Shoot fresh weight | 0.543 | 0.74 | 0..156 |
| Shoot dry weight | 0.539 | 0.762 | 0.098 |
| Root length | -0.563 | 0.165 | -0.703 |
| Root fresh weight | -0.257 | 0.744 | -0.431 |
| Root dry weight | -0.563 | 0.709 | 0.336 |
| SPAD | 0.544 | 0.408 | 0.121 |
| Root N content | 0.417 | 0.336 | 0.179 |
| Shoot N content | 0.282 | 0.16 | 0.105 |
| Root/Shoot | -0.968 | 0.012 | 0.215 |

**Table S4 Comprehensive factor Y value and N-tolerance ranking**

| Code | Y1 | Y2 | Y3 | Y | Order | Code | Y1 | Y2 | Y3 | Y | Order | Code | Y1 | Y2 | Y3 | Y | Order |
| --- | --- | --- | --- | --- | --- | --- | --- | --- | --- | --- | --- | --- | --- | --- | --- | --- | --- |
| 1 | -0.5451 | 2.5785 | 0.2496 | 0.4233 | 63 | 35 | -2.9657 | 3.4105 | -0.5533 | -0.5383 | 100 | 69 | -0.7942 | 4.7796 | 0.4846 | 0.8829 | 40 |
| 2 | -0.9692 | 3.1098 | 0.1884 | 0.3593 | 67 | 36 | -1.2128 | 3.5605 | -0.1899 | 0.3170 | 70 | 70 | -0.4204 | 3.4510 | 0.3842 | 0.7092 | 47 |
| 3 | -1.6303 | 3.5485 | 0.2745 | 0.1853 | 77 | 37 | -1.9430 | 2.7767 | 0.0363 | -0.1714 | 97 | 71 | -0.4620 | 3.5809 | 0.2891 | 0.7114 | 46 |
| 4 | -1.7364 | 3.4097 | 0.0598 | 0.0785 | 87 | 38 | -1.4683 | 2.8365 | -0.1240 | 0.0339 | 89 | 72 | -0.5332 | 3.3035 | 0.4501 | 0.6310 | 53 |
| 5 | -1.9847 | 3.8890 | 0.0193 | 0.0818 | 86 | 39 | -0.7179 | 3.6878 | -0.7236 | 0.5031 | 60 | 73 | 0.0857 | 4.1860 | 0.0967 | 1.0793 | 24 |
| 6 | -1.5354 | 3.3279 | -0.1560 | 0.1213 | 79 | 40 | -1.5690 | 3.8865 | -0.7384 | 0.1740 | 78 | 74 | -1.0658 | 4.5924 | 0.1038 | 0.6711 | 49 |
| 7 | -1.1513 | 3.2700 | 0.1925 | 0.3186 | 69 | 41 | -0.6284 | 3.2702 | -0.0222 | 0.5241 | 59 | 75 | 0.0123 | 3.4579 | 0.1117 | 0.8695 | 41 |
| 8 | -1.5580 | 3.4142 | 0.3354 | 0.1915 | 76 | 42 | -0.7095 | 2.5475 | -0.0371 | 0.3086 | 71 | 76 | -1.1860 | 2.5686 | 0.0791 | 0.1172 | 81 |
| 9 | -1.9269 | 3.1994 | 0.3425 | -0.0236 | 91 | 43 | -0.1320 | 5.0537 | 0.0991 | 1.1967 | 15 | 77 | -0.8860 | 3.1582 | 0.0980 | 0.3971 | 65 |
| 10 | -1.3714 | 3.6790 | 0.3523 | 0.3412 | 68 | 44 | 0.8904 | 3.5838 | 0.0226 | 1.2779 | 10 | 78 | -0.6361 | 3.2493 | 0.1865 | 0.5406 | 58 |
| 11 | -1.0455 | 3.7970 | 0.1584 | 0.4910 | 61 | 45 | 0.2051 | 3.6613 | 0.1728 | 1.0121 | 30 | 79 | -1.5882 | 4.2082 | 0.5846 | 0.4034 | 64 |
| 12 | -0.6847 | 3.7604 | -0.2114 | 0.5971 | 56 | 46 | 0.3129 | 3.7276 | 0.3047 | 1.0918 | 21 | 80 | -0.4382 | 4.3814 | 0.3458 | 0.9256 | 37 |
| 13 | -1.5734 | 3.0965 | 0.2230 | 0.0931 | 83 | 47 | 0.5096 | 4.0718 | 0.3310 | 1.2666 | 11 | 81 | -0.3210 | 3.7009 | 0.0925 | 0.7797 | 44 |
| 14 | -2.0518 | 3.8520 | 0.2265 | 0.0679 | 88 | 48 | 0.3267 | 3.1037 | 0.2586 | 0.9389 | 34 | 82 | -0.8188 | 3.7016 | 0.5582 | 0.6157 | 54 |
| 15 | -0.5208 | 4.0950 | 0.0969 | 0.7888 | 43 | 49 | -0.0839 | 4.8466 | 0.3158 | 1.1931 | 16 | 83 | -1.0555 | 4.2706 | 0.1565 | 0.6028 | 55 |
| 16 | -1.4315 | 3.6012 | 0.1510 | 0.2713 | 73 | 50 | -1.5841 | 5.1845 | 0.0971 | 0.5869 | 57 | 84 | -0.4834 | 4.2745 | 0.0329 | 0.8418 | 42 |
| 17 | -1.1495 | 3.9397 | -0.2662 | 0.4292 | 62 | 51 | -0.4811 | 4.7393 | -0.1803 | 0.9316 | 36 | 85 | -0.2113 | 2.9689 | 0.0503 | 0.6430 | 52 |
| 18 | -0.9377 | 3.5229 | -0.5188 | 0.3899 | 66 | 52 | -0.8151 | 4.1195 | 0.0588 | 0.6602 | 50 | 86 | -0.5859 | 3.7257 | 0.3432 | 0.6988 | 48 |
| 19 | -1.7168 | 3.7563 | -0.3707 | 0.1207 | 80 | 53 | 0.5261 | 4.5477 | 0.1343 | 1.3674 | 4 | 87 | -0.0278 | 4.2111 | 0.5119 | 1.0851 | 23 |
| 20 | -2.4276 | 3.7548 | 0.3027 | -0.1130 | 96 | 54 | -0.4123 | 5.1589 | 0.0100 | 1.0881 | 22 | 88 | 0.2781 | 4.6071 | 0.1906 | 1.2791 | 9 |
| 21 | -0.9871 | 4.4496 | -0.1203 | 0.6439 | 51 | 55 | -0.0057 | 3.8394 | 0.2319 | 0.9698 | 32 | 89 | -0.3901 | 4.6344 | 0.5975 | 1.0393 | 28 |
| 22 | -2.1467 | 3.9531 | -0.6017 | -0.0486 | 95 | 56 | 0.3478 | 4.8736 | 0.0288 | 1.3561 | 5 | 90 | -0.6838 | 4.9152 | 0.2391 | 0.9356 | 35 |
| 23 | -2.1971 | 4.3651 | -0.1133 | 0.0891 | 85 | 57 | 0.2326 | 3.8389 | 0.1916 | 1.0702 | 25 | 91 | 0.4605 | 4.1867 | 0.3944 | 1.2808 | 8 |
| 24 | -1.9946 | 4.2706 | -0.4407 | 0.1161 | 82 | 58 | -0.9769 | 5.7724 | 0.1505 | 1.0063 | 31 | 92 | 0.1301 | 4.7728 | 0.2733 | 1.2644 | 12 |
| 25 | -2.5161 | 4.2356 | 0.2110 | -0.0448 | 94 | 59 | -0.4384 | 4.6677 | -0.3444 | 0.9132 | 38 | 93 | -0.0259 | 4.6572 | 0.2549 | 1.1648 | 19 |
| 26 | -1.7596 | 3.4816 | -0.4031 | 0.0304 | 90 | 60 | 0.2880 | 4.1317 | 0.1824 | 1.1656 | 18 | 94 | -0.5149 | 4.7999 | 0.1245 | 0.9681 | 33 |
| 27 | -0.7235 | 2.5492 | -0.2287 | 0.2799 | 72 | 61 | 0.5361 | 4.6022 | 0.5223 | 1.4318 | 1 | 95 | 0.4915 | 4.6809 | 0.3343 | 1.4089 | 2 |
| 28 | -1.6922 | 2.8951 | -0.0171 | -0.0378 | 93 | 62 | 0.0760 | 3.7598 | 0.5267 | 1.0217 | 29 | 96 | 0.2713 | 4.2601 | 0.1524 | 1.1862 | 17 |
| 29 | -2.5091 | 2.8697 | 0.2139 | -0.3774 | 98 | 63 | 0.2107 | 3.6777 | 0.3769 | 1.0430 | 27 | 97 | 0.3303 | 3.6089 | 0.3029 | 1.0701 | 26 |
| 30 | -1.3203 | 3.0232 | -0.5621 | 0.0927 | 84 | 64 | -0.3375 | 4.1517 | 0.2339 | 0.9002 | 39 | 98 | 0.0101 | 4.9458 | 0.2532 | 1.2515 | 13 |
| 31 | -1.4229 | 3.5058 | -0.0374 | 0.2290 | 74 | 65 | 0.6424 | 3.7431 | 0.0570 | 1.2116 | 14 | 99 | 0.3671 | 4.5101 | 0.3610 | 1.3151 | 6 |
| 32 | -2.7434 | 2.9622 | 0.2086 | -0.4588 | 99 | 66 | -0.0784 | 3.0227 | 0.2347 | 0.7371 | 45 | 100 | 0.7352 | 4.3802 | 0.0185 | 1.4047 | 3 |
| 33 | -1.3949 | 3.4398 | -0.1244 | 0.2147 | 75 | 67 | 0.2789 | 4.5692 | 0.3651 | 1.2911 | 7 |  |  |  |  |  |  |
| 34 | -1.5332 | 2.8528 | -0.4744 | -0.0328 | 92 | 68 | -0.1288 | 4.6520 | 0.4215 | 1.1380 | 20 |  |  |  |  |  |  |
